# Promise of the NLRP3 Inflammasome Inhibitors in In Vivo Disease Models

**DOI:** 10.3390/molecules26164996

**Published:** 2021-08-18

**Authors:** Biswadeep Das, Chayna Sarkar, Vikram Singh Rawat, Deepjyoti Kalita, Sangeeta Deka, Akash Agnihotri

**Affiliations:** 1 Department of Pharmacology, All India Institute of Medical Sciences (AIIMS), Virbhadra Road, Rishikesh 249203, Uttarakhand, India; aakashagnihotri44@gmail.com; 2 Department of Clinical Pharmacology & Therapeutics, North Eastern Indira Gandhi Regional Institute of Health and Medical Sciences (NEIGRIHMS), Mawdiangdiang, Shillong 793018, Meghalaya, India; chayna_sarkar@hotmail.com; 3 Department of Psychiatry, All India Institute of Medical Sciences (AIIMS), Virbhadra Road, Rishikesh 249203, Uttarakhand, India; vikram.psyc@aiimsrishikesh.edu.in; 4 Department of Microbiology, All India Institute of Medical Sciences (AIIMS), Virbhadra Road, Rishikesh 249203, Uttarakhand, India; deep.micro@aiimsrishikesh.edu.in (D.K.); drsangeeta2009@gmail.com (S.D.)

**Keywords:** NOD-like receptors, NLRP3, inflammasome, inflammation, disease inhibitor

## Abstract

Nucleotide-binding oligomerization domain NOD-like receptors (NLRs) are conserved cytosolic pattern recognition receptors (PRRs) that track the intracellular milieu for the existence of infection, disease-causing microbes, as well as metabolic distresses. The NLRP3 inflammasome agglomerates are consequent to sensing a wide spectrum of pathogen-associated molecular patterns (PAMPs) and danger-associated molecular patterns (DAMPs). Certain members of the NLR family have been documented to lump into multimolecular conglomerates called inflammasomes, which are inherently linked to stimulation of the cysteine protease caspase-1. Following activation, caspase-1 severs the proinflammatory cytokines interleukin (IL)-1β and IL-18 to their biologically active forms, with consequent commencement of caspase-1-associated pyroptosis. This type of cell death by pyroptosis epitomizes a leading pathway of inflammation. Accumulating scientific documentation has recorded overstimulation of NLRP3 (NOD-like receptor protein 3) inflammasome involvement in a wide array of inflammatory conditions. IL-1β is an archetypic inflammatory cytokine implicated in multiple types of inflammatory maladies. Approaches to impede IL-1β’s actions are possible, and their therapeutic effects have been clinically demonstrated; nevertheless, such strategies are associated with certain constraints. For instance, treatments that focus on systemically negating IL-1β (i.e., anakinra, rilonacept, and canakinumab) have been reported to result in an escalated peril of infections. Therefore, given the therapeutic promise of an NLRP3 inhibitor, the concerted escalated venture of the scientific sorority in the advancement of small molecules focusing on direct NLRP3 inflammasome inhibition is quite predictable.

## 1. Introduction

Nucleotide-binding oligomerization domain NOD-like receptors (NLRs) are preserved pattern recognition receptors located in cell cytosol that track the intracellular milieu for the presence of infection, disease-causing microbes, as well as metabolic distresses. In humans, the NLR family is made up of 23 cytosolic proteins, and some 34 *nlr* murine genes have been determined. The usual segment structure of the NLR family members comprises of an amino-terminal effector part made up of a protein–protein interaction region, like the caspase-recruitment domain (CARD), pyrin domain (PYD), or Baculovirus inhibitor of apoptosis protein repeat (BIR) segment, a centrally positioned NOD domain and leucine-rich repeats associated with danger sensing at the carboxyl-terminal. Alterations at the N-terminal domain are employed for the subsequent assortment of NLR protein members. The biggest assortment incorporates the N-terminal PYD and has been christened the NLRPs. Certain members of the NLR family have been known to agglomerate into multimolecular structures called inflammasomes, which are inherently linked to the stimulation of the cysteine protease caspase-1. Following activation, caspase-1 severs the proinflammatory cytokines interleukin (IL)-1β and IL-18 into their biologically active entities, leading to the commencement of caspase-1-associated pyroptosis. IL-1β is an archetypic inflammatory cytokine implicated in multiple types of inflammatory maladies. Approaches to impede IL-1β’s actions are possible, and their therapeutic effects have been demonstrated; nevertheless, such strategies are associated with certain constraints. For instance, treatments that focus on systemically negating IL-1β (i.e., anakinra, rilonacept, and canakinumab) have been reported to subsequently result in an escalated peril of infections and are hence deemed improper for oral use. NLRP3 is the most common inflammasome sensor probed for its association in a multitude of conditions, such as sterile inflammation, infections, as well as uncommon genetic autoimmune syndromes (Table 1).

Given the therapeutic promise of an NLRP3 inhibitor, the concerted escalated venture of the scientific fraternity in the yesteryears towards the development of small molecules focusing on NLRP3 is quite predictable. However, incomplete comprehension of the steps leading to the NLRP3 inflammasome agglomeration and also insufficient understanding of the sensor crystal structure compound the odds of developing such inhibitor agents. Despite the fact that certain NLRP3 inflammasome antagonists have been developed and studied in preclinical protocols as well as cell-based assays, an NLRP3-specific inhibitor with therapeutic intent for humans has yet to be licensed. This review is intended to showcase the current developments with regard to promising NLRP3 inhibitors for clinical applications (Table 2).

## 2. NLRP3 Inflammasome Agglomeration

The NLRP3 inflammasome agglomerates are consequent to responding to a wide spectrum of pathogen-related molecular arrangements and damage-related molecular motifs. There is usually little cellular concentration of NLRP3, and in order to attain the critical threshold needed to spark caspase-1 activation, the canonical NLRP3 inflammasome stimulation hinges upon two crucial phases.

**Figure 1:**
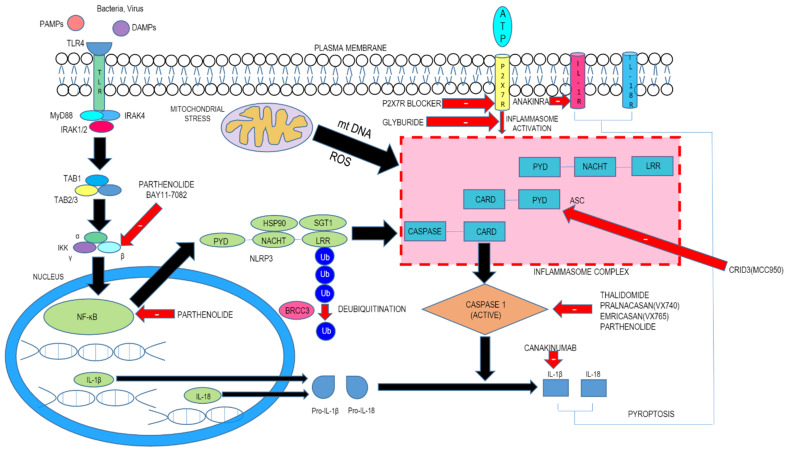
Inflammasome signaling cascade and inhibitors. The first step, termed as priming, leads to activation of nuclear factor kappa light chain enhancer of activated B cells (NF-kB) and various transcription factors following the involvement of pattern recognition receptors (PRRs) (TLR4). This engenders the expression of NLRP3 as well as pro-IL-1β. The second step comprises of signal (or trigger) detection that in turn governs the triggering mechanism of NLRP3 and the subsequent generation of the inflammasome.

The first step, termed priming, leads to activation of nuclear factor kappa light chain enhancer of activated B cells (NF-kB) and various transcription factors following the involvement of pattern recognition receptors (PRRs). This engenders the expression of NLRP3 as well as pro-IL-1β. The second step consists of signal (or trigger) detection that in turn governs the triggering mechanism of NLRP3 and the subsequent generation of the inflammasome. The NLRP3 NACHT region, which possesses ATPase property, is essential for the agglomeration of the NLRP3 inflammasome. Following stimulation and self-oligomerization of NLRP3, the PYD–PYD cooperation among NLRP3 and the inflammasome adaptor protein apoptosis-associated speck-like protein having a CARD (ASC) leads to the generation of speck-like entities, which serves as a scaffold for the engagement by contiguity of procaspase-1 via CARD–CARD interaction. Following autocatalytic enzyme cleavage and the subsequent generation of active caspase-1 (p10 and p20), the transformation of pro-IL-1β and pro-IL-18 into their biologically active forms occurs and gasdermin D–moderated pyroptotic cell lethality ensues. Since a wide array of signals finally culminates in NLRP3 inflammasome generation, it is believed that NLRP3 can detect downstream developments arising from the archetype trigger that produces disruption of cellular homeostasis, with subsequent inflammasome agglomeration (Figure 1). This disruption consists of alterations of ion flux (K^+^, Cl^−^, and Ca^2+^), reactive oxygen species (ROS) generation, and lysosomal injury.

## 3. Inhibitors of NLRP3 Inflammasome-Driven In Vivo Disease Models

### 3.1. Known Drugs

#### 3.1.1. Sulfonylureas (Like CRID3 (MCC950))

During the course of a phenotypic screen of ion channel inhibitors, Pfizer identified a compelling group of robust sulfonylureas capable of impeding IL-1β release, christened cytokine release inhibitory drugs (or CRIDs) [1]. These diarylsulfonylurea agents were patented in 1998 by Pfizer [39]. The patent failed to divulge the structure–activity relationships of these compounds, but comprehensive studies conducted for such agents (CP-424,174, CP-412,245, and CP-456,773) have distinctly established their capabilities. The last molecule, CP-456,773, also christened as CRID3 (or MCC950), is deemed to be a highly potent as well as a selective inhibitor of the NLRP3 inflammasome.

The study by Perregaux et al., 2001 [1] documented that nanomolar concentrations of these diarylsulfonylurea compounds impeded the cellular release of IL-1β. The compound CP-424,174 upon oral dosing in mice (ED50 around 15 mg/kg) also attenuated IL-1β secretion in vivo. The compounds failed to abrogate the secretion of IL-6 and TNFα and were presumed to be relatively selective [1].

The in vivo pharmacokinetic silhouette of MCC950, upon administration of a single dose of 3 mg/kg iv and 20 mg/kg po in C57BL/6 mice, displayed splendid enteral bioavailability of 68%, Cmax 25,333 ng/mL, AUC 163,410 ng h/mL, and half-life ~3 h [2]. Upon repeated administration of MCC950 for 5 days at 200 mg/kg p.o., no build-up was noted in mice serum; but extensive distribution data are unavailable [40]. In vivo concentrations of IL-1β following MCC950 administration to mice were attenuated by 50% with 0.4 mg/kg, 90% with 1.2 mg/kg, and above 90% with >4 mg/kg establishing the capability of this molecule [40]. MCC950 has been administered orally in high doses (such as 200 mg/kg doses) in several disease models, and no toxicological effects were encountered.

There is a concerted focus on the advancement of MCC950 as an intervention for the NLRP3-driven afflictions. MCC950 has been documented to impede the processing of IL-1β by caspase-1 in earlier studies [1]. Subsequently, Coll et al., 2015 [2] probed MCC950 in mouse and human macrophages and summarized its notable features. MCC950 has the capability to abrogate both canonical and non-canonical NLRP3 inflammasome signaling cascade stimulation and IL-1β secretion by impeding ASC oligomerization. Strikingly, MCC950 was bereft of any actions on AIM2, NLRC4, or NLRP1 inflammasome triggering [2,41,42]. Studies have been launched to completely dissect the process leading up to NLRP3 signaling blockade. MCC950 did not affect usual NLRP3 inflammasome triggering effects, such as efflux of potassium ions or signaling by calcium ions. There were no observed NLRP3 protein or ASC association inhibitions observed following MCC950 treatment [2]. Though intensely researched, the exact mode of action and binding characteristics of MCC950 remains elusive.

Another recent study has documented that MCC950 unswervingly associates with the NLRP3 NACHT domain’s Walker B motif and impedes ATP hydrolysis and assembly of the NLRP3 inflammasome [3]. Of late, a preprint paper at BioRxiv has documented that by engaging photoaffinity labeling as well as iBody technology, MCC950 associates with the NACHT domain of wild type NLRP3. The association was weakened in many CAPS-associated NLRP3 mutants, and furthermore, in two murine models of CAPS, MCC950 failed to abrogate the NLRP3-directed inflammatory pathophysiology. Such reports suggest that MCC950 may only be salutary in inflammation activated by wild-type NLRP3 protein and not in maladies activated due to CAPS-related NLRP3 mutants [43].

MCC950 was demonstrated to attenuate murine skin and pulmonary inflammation [40]. Cystic fibrosis (CF) pulmonary disease is featured by long-term infection with *Pseudomonas aeruginosa* with neutrophil-preponderant inflammation. The dearth of efficacious anti-inflammatory interventions for those afflicted with CF presents a stiff obstacle. NLRP3 inflammasome attenuation should provide an anti-inflammatory and anti-infective cover in CF. Selective NLRP3 inhibition in vivo with MCC950 abrogated IL-1β release in the CF mice lungs (*p* < 0.0001), with consequent indubitably repressed airway inflammation and augmented elimination of *Pseudomonas aeruginosa* (*p* < 0.0001) [44,45].

Several separate in vivo experiments in murine models of human multiple sclerosis (MS) exhibited that MCC950 mitigates the intensity of experimental autoimmune encephalo-myelitis (EAE) [2].

Amyotrophic lateral sclerosis (ALS) is an adult-onset, gradually advancing neurodegenerative disease arising out of the disintegration of motor neurons in the motor cortex, brain stem, and spinal cord. Current therapies for ALS are meager. As an illustration, the most extensively employed ALS therapeutic agent, riluzole, a glutamate release suppressor, extends survival by a meager 2–3 months on average. ALS microglia express NLRP3 and that pathological ALS proteins trigger the microglial NLRP3 inflammasome. Hence, impeding NLRP3 signaling activation should serve as a promising therapeutic methodology to abrogate microglial neuroinflammation and ALS disease advancement. A recent study established that ALS microglia contain NLRP3 and that pathological ALS proteins trigger the microglial NLRP3 inflammasome signaling cascade leading to IL-1β secretion. ALS microglial NLRP3 signaling cascade inhibition by MCC950 conspicuously attenuated both soluble and aggregated SOD1^G93A^ evoked secretion of IL-1β. Hence, MCC950 could serve as a promising therapeutic modality to abrogate microglial neuroinflammation and ALS disease advancement [46].

Oral administration of MCC950 has been demonstrated to salvage the dopaminergic system disintegration in a murine model of Parkinson’s disease (PD) [47]. Stimulation of the NOD-like receptor protein (NLRP3)-inflammasome signaling cascade is conjectured to assist with inflammatory reactions leading to brain impairment during ischemic/reperfusion (I/R) insult. Hence, it is tenable to surmise that treatment with MCC950, a selective NLRP3-inflammasome signaling cascade repressor, would invoke neuroprotection in murine models of transient middle cerebral artery occlusion (tMCAO). Ismael et al., 2018 [48] triggered focal cerebral ischemia for 60 min (tMCAO) with the subsequent intraperitoneal dispensation of MCC950 (50 mg/kg) or saline at 1 h and 3 h post-occlusion. After 24 h of I/R, mice were scrutinized for neurological outcome parameters and were euthanized in order to analyze infarct size as well as estimate NLRP3 inflammasome and apoptotic markers also. MCC950-exposed mice exhibited a sizable attenuation in infarction, edema, and Hb content (a marker of intracerebral hemorrhage) when correlated with saline controls along with the resolution of neurological insufficiencies. MCC950 attenuated the accumulation of NLRP3 inflammasome cleavage derivatives, caspase-1 and interleukin-1β (IL-1β) in the penumbral region. Such salutary actions of MCC950 were related with decreased TNF-α concentrations and poly(ADP-ribose) polymerase (PARP) and caspase-3 cleavage and corresponded with less phosphorylated NFκBp65 and IκBα concentrations. The total data hint that NLRP3-inflammasome signaling cascade repression with MCC950 has salutary actions in ischemic stroke models. More investigations into the therapeutic capability are imperative to establish if MCC950 use may serve as a potential representative agent for clinical trials.

Prospective studies are imperative to determine the precise promise of MCC950.

#### 3.1.2. Dual Action Sulfonylureas

On the basis of preceding awareness pertaining to antidiabetic agents used for managing Type 2 diabetes mellitus and utilizing the NLRP3 inflammasome suppressive effects of MCC950 (CRID3) in nanomolar concentrations, a fascinating assemblage of nine crossbreed compounds have been advanced [49].

These hybrid agents all exhibit nanomolar potency as suppressors of NLRP3 inflammasome stimulation, whereas a subassemblage (consisting of crossbreed molecular entities incorporating acetohexamide, glibenclamide, gliquidone, glimepiride, and glisoxepide) have been observed to facilitate secretion of insulin akin to parent sulfonylurea compounds [49]. In vivo study results have yet to be published, but several uses may be envisaged. During the advancement of type 2 diabetes mellitus, it has been recognized that the β-cell mass in the pancreas is reduced. This is the reason for the dwindling capability of sulfonylurea drugs in this affliction. NLRP3-evoked IL-1β secretion has been documented to be responsible for pancreatic β-cell death. From a logical angle, such molecules with a dual mode of action (such as those obviating β-cell mortality as well as provoking secretion of insulin) could possibly bring about beneficial actions. As a result of abrogation of NLRP3 inflammasome stimulation by such engendered crossbreed molecules, many afflictions of the likes of neuroinflammation, coronary atherosclerosis, nephropathy, and wound healing might be mitigated to a large extent.

BIIB093 is an intravenous (IV) formulation of glibenclamide (also christened as glyburide). Glibenclamide administered by the intravenous route as a therapeutic intervention for ischemic stroke has been undergoing testing in clinical trials due to its suppression of sulfonylurea receptor-1-transient receptor potential melastatin-4(SUR1-TRPM4) ion channels which mitigates oedema and hemorrhagic transformation [50]. SUR1-TRPM4 channels have been demonstrated to be increased in numbers and sensitivity subsequent to decreased blood flow in hypoperfused endothelial cells, neurons, and glia, and has been related to cytotoxic edema, BBB disruption, and development of blood vessel-associated edema [51]. Supplementary studies have also shown that actuated microglia in the ischemic lesion core contain Sur1-Kir6.2 channels [52]. In many animal studies engaging both short-term and long-term models of middle cerebral artery occlusion (MCAO) and thromboembolic representation of ischemic stroke, it has been demonstrated that administration of glibenclamide remarkably attenuates ischemic infarct size and hemispheric distension, even when given many hours post-stroke commencement [53,54,55]. Scrutiny of long-term outcomes of glibenclamide treatment has demonstrated notable benefits in sensorimotor and cognitive attributes, observed a month after the onset of stroke [56]. The response to glibenclamide has been probed in rodent models of lethal stroke associated with malignant cerebral edema, where it markedly mitigated edema and death rate [53,57]. The latest safety and efficacy of intravenous glyburide on brain swelling after a large hemispheric infarction (GAMES-RP) clinical trial established a massive attenuation in cerebral edema and midline movement without an effect on the results [58]. The industry-sponsored Phase 3 Study to Evaluate the Efficacy and Safety of Intravenous BIIB093 (Glibenclamide) for Severe Cerebral Edema Following Large Hemispheric Infarction (CHARM) clinical trial examining a comparable hypothesis involving the sulfonylurea glibenclamide is afoot (ClinicalTrials.gov: NCT0286495) [59,60]. Subsequent to hemorrhagic stroke engendering tissue injury, NLRP3 inflammasome stimulation has been chronicled [61]. The dual action glyburide-MCC950 hybrid may be conjectured to afford even more salutary effects than glyburide in the therapy of stroke.

#### 3.1.3. Sulfonamides (JC-21, JC-171)

The synthesized sulphonamide 16673-34-0, christened as JC-21, has been experimentally noted to be an NLRP3 inflammasome signaling inhibitor [62]. It has been found to offer beneficial effects in disease models of ischemia–reperfusion injuries and myocardial infarction in mice [62,63,64].

The hydroxysulfonamide analog of JC-21, termed JC-171, was designed and synthesized in order to address solubility concerns with JC-21 [65]. At doses of 100 mg/kg, JC-171 was found to prevent LPS-evoked IL-1β release in vivo in murine models. At doses of 100 mg/kg (administered ip every alternate day), JC-171 has been observed to notably impede onset as well as mitigate disease symptoms in an EAE disease model in mice. Subsequent to disease onset in the murine EAE model, both MCC950 and JC-171 (10 mg/kg ip on alternate days) exhibited similar capability in impeding disease progression.

#### 3.1.4. Glitazones (CY-09)

CY09 was selected via structure–activity relationship observations of C172, a hit determined by examining a bioactive library. Jiang et al., 2017 determined this effective and direct suppressor of NLRP3, which exhibited remarkable suppression of NLRP3 inflammasome in vivo in murine models and ex vivo in human cells [4]. CY09 shares a chemical resemblance with CFTR(inh)-172(C172), which impedes the cystic fibrosis transmembrane conductance regulator (CFTR) channel [66]. However, CY-09 is devoid of CFTR-inhibitory actions [67]. In bone marrow-derived macrophages (BMDMs) kindled with LPS, CY-09 dose-dependently obtunded the ATP, monosodium urate (MSU), and nigericin-evoked stimulation of caspase-1 and consequent secretion of IL-1β. Its suppressant action is independent of signal 1 and NLRP3 posttranslational alteration (ubiquitination). Mechanistically, it directly collaborates with the NLRP3 Walker A motif to exclude the ATP association of NLRP3, but NLRP1, NLRC4, RIG-1, or NOD2 are unaffected. Notably, CY09 binding efficacy has been reported to be curtailed when the Walker A motif was altered, implying that CY09 possesses an association site in this pattern [4].

Most importantly, CY-09 exhibited a promising pharmacokinetic profile and demonstrated good oral bioavailability, safety, and stability. CY-09 had favorable pharmacokinetic characteristics [4], such as stability in plasma and no inhibitory effect on the hERG ion channels at concentrations ≤10 μM. The five major cytochrome P450 isozymes were also spared of any inhibitory effects. The in vivo half-life bioavailability and AUC of CY-09 in mice have been documented as 2.4 h, 72%, and 8232 (h ng)/mL, respectively, which is useful in the assessment of its effects in relevant disease models in mice.

CY-09 demonstrated exceptional preventive or therapeutic prowess in mice models of gout, T2D, and cryopyrin-associated periodic syndromes (CAPS). CY-09 established itself as a remarkably efficacious agent in animal models of monosodium urate (MSU)-evoked peritoneal inflammatory disorder as well as sensorineural deafness and recurrent hives (Muckle–Wells) syndrome. In these animal models, both CY-09 and MCC950 produced similar efficacies at 40 mg/kg ip dose. In another study, CY-09 administration was found to result in salutary effects in diabetic mice with a metabolic disorder. MCC950 was, however, not employed as a comparator drug in that study. Thus, CY-09 has become an attractive lead for further research in the area of NLRP3 inhibitors, which interact with the ATP site.

Moreover, confirmatory studies are imperative to broaden its full potential [4].

#### 3.1.5. Edaravone

Edaravone has now been inducted as a generic treatment strategy for amyotrophic lateral sclerosis (ALS) (certified for use in 2015 and 2017 in Japan and the USA, respectively) and stroke (certified for use in 2011) [68,69]. The precise mechanism of edaravone’s action in inhibiting NLRP3 inflammasome inhibition is still fuzzy. However, it is known to possess antioxidant, free radical scavenging, as well as neuroprotective effects. It is surmised that scavenging reactive oxygen species (ROS) edaravone impedes NLRP3-evoked IL-1β processing and release from microglia treated with amyloid-β [5].

Intracerebral hemorrhage (ICH) is a prominent reason for disability and mortality in adults, for which there is a dearth of practical therapies in a realistic sense. Edaravone has displayed its neuroprotective properties subsequent to ischemic stroke. However, its probable mode of action for these effects post-ICH are fuzzy at best. Miao et al., 2020, reported in a recent study that edaravone administration led to neuroprotection post-ICH in part by suppressing IL-1β, caspase 1, and NF-kB-dependent NLRP3 inflammation signaling in microglia in a rat model. This paves the path for a novel affirmation for clinic usage of edaravone after ICH [6]. Edaravone has been known to produce certain side effects like skin inflammation, hypersensitivity, and gait disturbance [70].

These studies concerning edaravone are only foundational in nature, and more comprehensive scrutiny is imperative to completely demonstrate its actions on the NLRP3 inflammasome signaling pathway.

#### 3.1.6. Antidepressants

Mounting scientific documentation suggests the engagement of inflammatory mechanisms and pro-inflammatory cytokines in the causation of major depressive disorder (MDD), as well as refractoriness to antidepressant intervention [71]. Heightened interleukin (IL)-1β and IL-18 signaling has been determined to propagate resistance to selective serotonin reuptake inhibitors (SSRIs) in certain entities of depression [72]. Of late, the NLRP3 inflammasome has been reported to be activated in blood mononuclear cells (BMCs) in MDD patients and lipopolysaccharide-induced mice with MDD-like behaviors, prompting the thought that this could possibly be a novel aspect of MDD pathogenesis [73,74]. The NLRP3 inflammasome activation mediated IL-1β release and neuroinflammation has recently been implicated with great gusto in major depressive disorder (MDD) pathogenesis. This has sparked serious efforts at therapeutic modulation of these pathways [75]. A collection of antidepressant agents employed in clinical practice (to counter MDD) have been probed for their anticipated action on NLRP3 inflammasome [76].

Alcocer-Gomez et al., 2017 [76] administered a chemically diverse group of antidepressant molecules (viz., fluoxetine, paroxetine, mianserin, mirtazapine, venlafaxine, desvenlafaxine, amitriptyline, imipramine, and agomelatine) in their experiments. All these agents led to a 50–60% decrement in IL-1β and IL-18 release when probed in an LPS-kindled THP-1 cell line employing ATP as the NLRP3 inflammasome stimulating agent at concentrations of 1 μM. NLRP3 expression was also abrogated by these molecules. In comparison to the other analogs, agomelatine, mirtazapine, and fluoxetine were noted to be marginally more efficacious.

Employment of the forced swimming test in a mice depression model, all the animals exhibited enhancement of IL-1β processing and release, which was mitigated by antidepressant administration [76].

A diagnosed set (by personal interview) of 214 MDD patients (aged 18–60) were recruited in a study by Alcocer-Gomez et al., 2017 [76]. Nine specific antidepressant drugs (fluoxetine, paroxetine, mianserin, mirtazapine, venlafaxine, desvenlafaxine, amitriptyline, imipramine, and agomelatine) were investigated for their effects on the NLRP3 inflammasome in humans [76]. The serum of all antidepressant-treated patients exhibited decrement of IL-1β and IL-18 levels in concert with decreased presence of NLRP3 mRNA in mononuclear cells in blood when compared to untreated controls. Significant upregulation of genes typical of autophagy (BECLIN and MAP-LC3) were indicative of the fact that heightened functioning of autophagy pathways may prove salutary. In autophagy-knockout mouse embryonic fibroblast cells, in vitro, no antidepressant could repress NLRP3 inflammasome signaling pathways. In line with this assumption, an increment of LC3B-11 (autophagy readout) and no decrement in cleaved caspase-1 has been noted. A powerful decrement in ATP-kindled NLRP3 inflammasome signaling with antidepressant treatment has been observed in wild-type murine embryonic fibroblast cells. This was associated with an increment of LC3B-11 and a decrement of split-processed caspase-1. Such comprehensive findings have opened up the exciting probability that NLRP3 inflammasome may serve as a biomarker for therapeutic efficacy and hence facilitate the choice of medications.

Strikingly, an extensively used antidepressant, fluoxetine, indubitably represses ROS–PKR (double-stranded RNA-dependent protein kinase)–NLRP3 signaling cascades located inside macrophages and microglia. Moreover, fluoxetine represses NLRP3 inflammasome stimulation in macrophages as well as the hippocampus in mice models of long-term mild-stress, implying an encouraging therapeutic scenario for therapy of NLRP3 inflammasome-associated depression [77].

#### 3.1.7. Anthranilic Acid NSAIDs

In addition to their widespread use as cyclo-oxygenase (COX)-inhibiting non-steroidal anti-inflammatory agents (NSAIDs), certain fenamic acid derivatives have been documented in the contemporary research literature to produce actions on NLRP3 inflammasomes [7]. LPS-kindled immortal mouse BMDM cells have been exposed to four such specific molecules employed clinically (viz., mefenamic acid, flufenamic acid, meclofenamic acid, and diclofenac). Upon stimulation of the cells with ATP, these agents repressed IL-1β release. In the assays, meclofenamic acid (with an IC50 around 25 μM) was noted to be the most potent inhibitor. The structurally unconnected NSAID, ibuprofen, did not exert any effect. Analogous results were noted upon administration of MSU as an NLRP3 stimulator. Probing the fenamates employing NLRC4 evoking impulse *S. typhimurium* or AIM2 impulse dsDNA demonstrated that the agents failed to suppress these inflammasomes and hence possessed a hint of selectivity. Incidentally, ASC speck formation was also suppressed by the fenamate derivatives.

The salutary effects of mefenamic acid were studied in in vivo models of Alzheimer’s disease [41]. The first therapeutic model exhibited a protective effect upon prophylactic intervention when administered mefenamic acid (5 mg/kg/day, ip) in rats pretreated with the intracerebroventricular injection of soluble oligomeric amyloid β1–42. The other therapeutic model tested 13–14-month-old 3× TgAD transgenic mice in which mefenamic acid was administered round the clock (25 mg/kg/day) with the aid of an osmotic mini pump [41]. The intervention fully abolished neuroinflammation with simultaneous attenuation in stimulated microglia and IL-1β. This observation is promising as these agents are regularly used in clinical settings and hence may be promptly repurposed.

#### 3.1.8. Arsenic Trioxide and Other Arsenic Compounds

In humans, inflammasome complexes function as crucial molecular targets of arsenic. In the human monocyte cell line, THP-1 and murine bone marrow-derived macrophages, As_2_O_3_ suppresses NLRP3 inflammasome stimulation and consequent IL-1β and IL-18 release by acting on promyelocytic leukemia (PML) [7,8]. Furthermore, As_2_O_3_ represses NLRP1, NAIP5/NLRC4 inflammasomes in the same cell subsets [78]. However, NaAsO_2_ facilitates IL-1β and IL-18 release through AIM2 inflammasome activation in the human keratinocyte cell line and mouse skin tissue [79]. Additionally, the inflammasome NALP2 polymorphism is related to arsenic-induced skin lesions in humans [80]. Hence, As(III) produces bidirectional modulation on inflammasomes, which is possibly tissue dependent [81].

NLRP3 inflammasome brings about the sterile inflammatory reaction provoked by tissue injury and influences the causative pathology of myocardial ischemia–reperfusion injury [64,82,83]. Abrogating inflammasome stimulation by genetic method conspicuously attenuates infarct progress and myocardial fibrosis and malfunction [64,82].

Finally, the fact that arsenic abrogates inflammasome signaling and the consequent IL-1β synthesis in human macrophages acts as a pointer that arsenic may restrict chronic inflammation in intense inflammasome-associated disease [84].

#### 3.1.9. Colchicine

Kajikawa et al., 2019 [85] recorded that short-term use of low-dose colchicine notably mitigated endothelial inflammation with attenuation of serum high-sensitivity C-reactive protein (hs-CRP) levels in CAD patients. This observation provides a novel role of colchicine in anti-inflammatory atheroprotection, but it is imperative to further scrutinize the underlying pathways.

Colchicine has been reported to usher in beneficial actions in multiple afflictions via NLRP3 suppression. Martinez et al., 2015 [9] identified that short-term colchicine treatment efficaciously attenuated the expression levels of IL-1β, IL-6, and IL-18 by abrogating the NLRP3 inflammasome activation cascade in ACS patients. Otani et al., 2016 [10] found that colchicine ameliorated NSAID-evoked small bowel damage via suppression of NLRP3 inflammasome signaling activation and ILβ maturation. These results act as a pointer that colchicine may provide salutary anti-inflammatory effects by blunting NLRP3 inflammasome activation.

NLRP3 activity is positively correlated with the concentrations of CRP expression and inflammatory reaction. Li et al., 2016 [86] characterized that the activation of NLRP3 inflammasome incremented CRP and MCP-1 concentrations, whereas NLRP3 knockdown attenuated CRP and MCP-1 concentrations in HUVECs. Ridker et al., 2016 [87] ascertained that NLRP3 stimulation bolstered the generation of IL-1β and IL-6 along with up-regulation of CRP expression, which intensified atherogenesis. These results express that colchicine could mitigate endothelial inflammation via the NLRP3/CRP signaling pathway. However, further prospective testing is imperative to corroborate this result. Inflammation plays a pivotal role in clinical expression and complexities associated with acute coronary syndromes (ACS). Colchicine, a widely used agent for gout, has lately evolved into a novel therapeutic choice in cardiovascular medicine due to its anti-inflammatory effects. Tong et al., 2020 [88] ascertained the potential utility of colchicine use in ACS patients in a multicenter, randomized, double-blind, placebo-controlled trial executed at 17 hospitals in Australia providing acute cardiac care service. However, it was reported with disappointment that concurrent use of colchicine with standard medical therapy failed to significantly produce salutary cardiovascular results at 12 months in ACS patients and was linked to elevated mortality.

Colchicine for oral administration (capsule/tablet/liquid) is presently FDA endorsed for the prevention and management of gout attacks in adults afflicted with gout and familial Mediterranean fever (FMF) (https://www.fda.gov/drugs/postmarket-drugsafety-information-patients-and-providers/colchicine-marketed-colcrys-information, accessed on 25 May 2021). Non-licensed utilities of colchicine are multiple and are inclusive of afflictions such as acute calcium pyrophosphate (CPP) arthritis (pseudogout), sarcoid and psoriatic arthritis, Behcet’s disease, and pericarditis, and of late, experiments have demonstrated colchicine’s capability in obviating leading cardiovascular untoward effects in patients who experienced a recent myocardial infarction [89]. Though being in clinical use for almost 2000 years, novel therapeutic applications of colchicine, farther than gout [90], is receiving consideration. Colchicine trials to counter inflammation in COVID-19-affected patients have experienced scant consideration. Of late, about 34 colchicine clinical trials are advancing in the intervention of SARS-CoV-2 infection and have been enlisted in clinicaltrials.gov (https://clinicaltrials.gov/ct2/results?cond=COVID&term=colchicine&cntry=&state=&city=&dist=, accessed on 25 May 2021). In a murine myocardial infarct model, with doses comparable to those employed in humans (0.1 mg/kg), colchicine attenuated the activation of NLRP3 inflammasome constituents over and above the decrement in inflammatory mediators [9,91,92]. Otani et al., 2016 [87] probed the actions of colchicine on the non-steroidal anti-inflammatory drug (NSAID)-evoked small intestinal damage and stimulation of the NLR family pyrin domain-containing 3(NLRP3) inflammasome. Colchicine administration suppressed indomethacin-evoked small intestinal damage to the tune of 86% (at a dose of 1 mg/kg) and 94% (at a dose of 3 mg/kg) as determined from the lesion index 24 h following indomethacin use. Colchicine abrogated protein expressions related to cleaved caspase-1 as well as mature IL-1β, sparing the mRNA expressions of NLRP3 and IL-1β. The protective actions of colchicine in this model were abrogated by the administration of a dose of recombinant IL-1β (0.1 μg/kg). The indomethacin-induced small intestinal injury was decreased by 77%, as noted from the scrutiny of the lesion index in NLRP3−/− mice, and colchicine therapy could not abrogate small intestinal injury in NLRP3−/− mice. These outcomes establish that colchicine obviates NSAID-evoked small intestinal damage by repressing NLRP3 inflammasome stimulation. A clinical trial is imperative to establish the effectiveness of colchicine in treating NSAID-evoked small intestinal injury. Colchicine has also been noted to alleviate NLRP3 inflammasome activity in a murine model of Coxsackie virus B3-induced myocarditis [93].

Since colchicine bolsters AMPKα phosphorylation and suppresses the stimulation of caspase-1 and the secretion of IL-1β during inflammasome activation, AMPK activation has been associated with modulating monosodium urate (MSU) crystal-induced inflammasome activation [94,95].

#### 3.1.10. Metformin

Metformin has been demonstrated to decrease the expression of NLRP3 as well as activation of the NLRP3 inflammasome signaling pathway. The inhibitory process is achieved via the augmented levels of both adenosine monophosphate-activated protein kinase (AMPK) as well as protein phosphatase 2A (PP2A) [11]. Furthermore, metformin decrements glucose levels, thereby potentially contributing to the attenuation of NLRP3 inflammasome stimulation. In vitro analysis dissected the decreased activation rate of NLRP3 inflammasome in the murine ApoE−/− model and impeded atherosclerotic process in diabetes [96].

Periodontitis is one of the most frequent chronic inflammatory diseases occurring in human periodontium as a result of dental bacterial infections, with consequent damage to tooth-supporting structures ultimately culminating in tooth loss. Tan et al., 2020 [12] established that metformin elicited protective effects on *Porphyromonas gingivalis (P. gingivalis)* induced inflammatory process and impeded the stimulation of NLRP3 inflammasome signaling as well as IL-1β and IL-18 release. In addition, NF-κB signaling pathway associated genes and TNF-α linked genes have been implicated in the anti-inflammatory actions of metformin. Therefore, for the prevention and treatment of *P. gingivalis* associated periodontal diseases, the NLRP3 inflammasome inhibitory action of metformin could be employed. Additional animal studies need to be conducted to decode the exact mechanism of NLRP3 inflammasome signaling in periodontitis and also the salutary action of metformin in repressing NLRP3 inflammasome activation in vivo before conducting more experiments in human subjects.

AMPK is known to modulate NLRP3 inflammasome stimulation. Metformin has been an extensively employed antidiabetic agent for type 2 diabetes which may probably assume a cardioprotective role via numerous pathways. Metformin exerts agonistic actions on AMP-activated protein kinase (AMPK), thereby blocking mitochondrial complex I. The NLRP3 inflammasome has been established to be stimulated in diabetic cardiomyopathy (DCM). However, the precise mechanisms by which metformin modulates the NLRP3 signaling cascade in DCM remains nebulous. There have been reports that AMPK can suppress NLRP3 by initiating autophagy. Yang et al., 2019 [97] studied the actions of metformin on the NLRP3 cascade in elevated glucose-administered cardiomyocytes as well as diabetic mice and subsequently probed the actual processes by which metformin affects the AMPK/mTOR signaling cascade. These data enable one to acquire a novel insight into metformin’s actions in DCM regulation and concluded that metformin does indeed elicit cardioprotective as well as anti-inflammatory actions by stimulating AMPK/autophagy and consequently suppressing the NLRP3 inflammasome in DCM.

Skin wound healing is a demanding obstacle, specifically in geriatric and diabetic patients, which are tough to heal, and is a massive public health concern. Metformin facilitated wound healing and augmented angiogenesis, affording a supplementary anti-inflammatory cover via the modulation of the AMPK/mTOR/NLRP3 inflammasome signaling cascade in which NLRP3, which bolstered M2 macrophage polarization, led to acceleration of wound healing. These data enable a peek into the novel molecular mechanism associated with metformin administration and its therapeutic promise in wound healing [98].

In those afflicted with type 2 diabetes, enhanced NLRP3 inflammasome stimulation and processing of IL-1β were notably repressed by treatment with the antidiabetic drug metformin via AMPK stimulation [99].

In another study, AMPK stimulation through metformin administration was associated with inhibition of hyperalgesia related to NLRP3 inflammasome activation and high levels of IL-1β and IL-18 in mice; moreover, there was a noticeable improvement in the clinical parameters in patients suffering from fibromyalgia, a chronic pain disorder [100].

The immune system exerts a vital and central role in tumor cell differentiation, proliferation, angiogenesis, apoptosis, invasion, and metastasis. By modulating the AMPK/mTOR signaling cascade with consequent suppression of NLRP3 inflammasome activation, metformin is capable of impeding cancer progression in a promising fashion [98]. Moreover, metformin exposure has also been reported to facilitate a notable decrement in ROS generation in CD11+ MDSCs and TAMs in neoplasms [101] and repression of the progression of prostate cancer by obstructing the infiltration of TAMs via the inhibition of COX2/PGE2 cascade, eliciting that intervention with a combination of standard therapy + metformin could provide enhanced treatment efficiency [102].

#### 3.1.11. Liraglutide

Liraglutide, a glucagon-like peptide-1 (GLP-1) analogue that has currently become the frontline intervention for type 2 diabetes mellitus (T2DM), has also been determined to attenuate fatty hepatic degeneration. Zhu et al., 2018 [13] performed experiments to probe whether liraglutide is of benefit in high-fat diet-evoked murine non-alcoholic fatty liver disease (NAFLD) via repression of the hepatic NLRP3 inflammasome. Consequent upon intraperitoneal injection of liraglutide every day (dosed at 0.6 mg/kg body weight) for four weeks, the liver, liver/body weight, serum levels of ALT, AST, total cholesterol, triglycerides, and LDL were markedly attenuated in a murine model of high-fat diet-induced NAFLD. The hepatic steatosis in sections of H&E and Oil Red O staining was also notably decreased following exposure to liraglutide. The levels of NLRP3 inflammasome components (such as NLRP3, ASC, and caspase-1) in the liver of mice following administration of liraglutide were decreased considerably. In vitro experiments reported that mitochondrial malfunction in Kupffer cells evoked by palmitic acid was decreased, and the protein concentrations of NLRP3, ASC, and caspase-1 were also attenuated significantly. These outcomes established that liraglutide was capable of alleviating high-fat diet-induced hepatic steatosis by suppressing NLRP3 inflammasome stimulation, providing a lead that liraglutide is a potential agent which may reverse the pathological characteristics of NAFLD [13].

#### 3.1.12. Statins

Statins are necessary for obviating and treatment of cardiovascular afflictions by repressing cholesterol synthesis. However, the salutary actions of statins in cardiovascular afflictions has also been conjectured to accrue out of their anti-inflammatory mechanisms. Atorvastatin, a 3-hydroxy-3-methyglutaryl coenzyme A (HMG-CoA) repressor, markedly attenuated the levels of NLRP3, caspase-1, and IL-1β in PMA-evoked THP-1 cells. Additionally, the NF-κB suppressor attenuated the levels of inflammatory cytokines in inflammatory cells. It was advanced that the stimulation of the NF-κB signaling cascade was engaged in the modulation of the NLRP3 inflammasome [14,15]. Hence, atorvastatin exerts an anti-inflammatory action by suppressing the PMA-evoked THP-1 monocyte through the TLR4/MyD88/NF-KB signaling cascade. In vitro and in vivo exposure with simvastatin accounted for perceptibly lesser expression levels in response to activation with cholesterol crystals (CCs). Simvastatin suppressed levels of IL-1β, peripheral blood mononuclear cells (PBMCs), and CCs and subsequently had salutary action on those suffering from cardiovascular afflictions [103].

#### 3.1.13. SGLT-2 Inhibitors (Dapagliflozin)-P2Y12 Antagonist (Ticagrelor)

Anti-diabetic agents are chiefly employed to manage hyperglycemic disorders in those afflicted with diabetes mellitus. Such agents also facilitate diabetic patients to maintain their malady under check and also attenuate the probability of diabetes-related complexities. Contemporary scientific documentation proposes that a few antidiabetic drug groups obstruct the NLRP3 inflammasome signaling pathway and evince anti-inflammatory actions in diabetic nephropathy patients as well as experimental animal models [104]. Inhibitors of sodium-dependent glucose transporter 2 (SGLT-2) have been administered to decrement blood glucose levels in patients suffering from type 2 diabetes mellitus. This class of drugs useful in diabetes impedes glucose reabsorption from S2 and S3 segments of kidney proximal tubules and hence decreases glucose levels in an insulin-independent fashion [105]. Reports have established that the SGLT-2 inhibitor dapagliflozin extenuates inflammation-evoked renal damage and glomerulosclerosis in diabetic kidneys by ameliorating NLRP3 inflammasome stimulation. A decrement in mRNA expression levels of ASC, caspase-1, IL-6, IL-1β, and TNF-α have also been established [16]. SGLT-2 inhibitors are believed to reduce glomerular hyperfiltration, oxidative stress as well as inflammation in diabetic nephropathy. Empagliflozin (a different SGLT-2 inhibitor) has also been determined to impede the kindling of NLRP3 inflammasome and decrements downstream inflammatory signaling in the diabetic kidneys in another study [17]. Nevertheless, further research is imperative to determine the preliminary safety and efficacy data of SGLT2 inhibitors in the progression of diabetic kidney disease.

Ticagrelor, a P2Y12 receptor antagonist, and dapagliflozin, an SGLT2 inhibitor, repress the stimulation of the NLRP3 inflammasome. The anti-inflammatory actions of dapagliflozin are associated with the activation of AMPK. Additionally, ticagrelor has the capability to stimulate AMPK. Chen et al., 2020 [18] probed whether dapagliflozin and ticagrelor possess supplementary actions in impeding the progression of diabetic cardiomyopathy in mice suffering from T2DM. Eight-week-old BTBR and wild-type mice were administered no drug, dapagliflozin (1.5 mg/kg/day), ticagrelor (100 mg/kg/ day), or their combination for a duration of 12 weeks. Cardiac activity was determined with the aid of echocardiography, and heart tissue samples were evaluated for fibrosis, apoptosis, qRT-PCR, as well as immunoblotting. Dapagliflozin as well as ticagrelor impeded the progression of diabetic cardiomyopathy as established by recovery of left ventricular endsystolic and end-diastolic volumes as well as left ventricular ejection fraction, which was enhanced further by concomitant administration of the combination. Both agents impeded the stimulation of NOD-like receptor 3 (NLRP3) inflammasome and fibrosis. The outcome with the drug combination was notably better than monotherapy (with each drug alone) on levels of myocardial tissue necrotic factor α (TNFα) as well as interleukin-6 (IL-6), indicating an additive outcome. Moreover, the administration of the drug combination was associated with a larger effect on levels of ASC, collagen-1, and collagen-3 mRNA expression when compared with monotherapy (with each drug alone).

#### 3.1.14. Melatonin

Melatonin is capable of suppressing levels of IL-1β, NLRP3, cleaved caspase-1, and a multitude of proteins connected with the aortic endothelium. It has also been determined that melatonin activates mitophagy and NLRP3 inflammasome activation abrogation via the Sirt3/FOXO3a/ Parkin signaling pathway [106].

#### 3.1.15. IL-1/ IL-1R-Targeted Agents: Anakinra, Canakinumab, and Rilonacept

Upon triggering of the NLRP3 inflammasome activation, the pro-inflammatory mediator IL-1β is generated and secreted out of the inflammatory cells. IL-1β is a common cytokine associated with NLRP3-driven maladies [107]. Hence, IL-1β could be viewed as the principal target in impeding NLRP3-triggered inflammation. Generally, many agents that focus on the IL-1/IL-1 receptor (IL-1R) axis have been employed in clinical trials. Currently, three IL-1/ IL-1R-targeted molecules have been identified with a therapeutic promise: the IL-1 receptor antagonist anakinra, the soluble camouflage receptor rilonacept, and the neutralizing monoclonal anti-IL-1β antibody, canakinumab [108], out of which canakinumab and anakinra are currently used clinically [109]. Additionally, a monoclonal antibody focusing on IL-1R and a counteracting anti-IL-1α antibody are presently undergoing clinical trials [110]. Canakinumab directly focuses on IL-1β owing to its selective immune appreciation, whereas anakinra represses the IL-1/IL-1R signaling cascade by inhibiting IL-1α/β and IL-1R interaction. Obviously, varied mechanisms of action will produce varied clinical effects. Anakinra was chiefly advanced to intervene in NLRP3-driven maladies such as CAPS and rheumatoid arthritis. However, the convenience of anakinra in clinical settings is curbed owing to its brief plasma half-life (4–6 h) and the difficulty for patients to continue the treatment [111]. On the other hand, canakinumab has an extended half-life (26 days), which makes it advantageous for patients to continue the treatment [110]. Certainly, pharmacological repression of IL-1 could markedly mitigate the intensity of gout in patients who have been nonresponsive to regular interventions or in cases where NSAIDs, colchicine, or glucocorticoids are contraindicated. In a recent development, a research group has found that continuous administration of canakinumab could notably mitigate the frequency of atherosclerotic plaques in those with elevated C-reactive protein (CRP) concentrations [112]. Furthermore, with canakinumab administration, the incidence of arthritis and gout in patients was also markedly attenuated [113]. Collectively, these outcomes demonstrate that interventions grounded on the targeting of the IL-1/IL-1R signaling cascade provide an alluring alternative for the management of NLRP3-driven afflictions. However, unavoidable challenges still persist. Firstly, all three therapies have to be delivered parenterally, and so the modality is uncomfortable and painful [114]. Secondly, neglecting their origin, blockade of total IL-1β signaling is bound to heighten the probability of infection. Furthermore, mechanisms that only impede the IL-1/IL-1R signaling cascade will have lesser effectiveness in the intervention of NLRP3-driven afflictions, which are also facilitated by many pro-inflammatory cytokines, such as IL-18 and HMGB1 [115]. Hence, although targeting of the IL-1/IL-1R axis has demonstrated clinical promise under a few situations, the identification of specific and direct NLRP3 inhibitors to intervene in NLRP3-associated diseases continues as an exciting aim. Future inhibitors have the promise of enhanced specificity and fewer side effects.

#### 3.1.16. Allopurinol

Present-day studies indicate that NLRP3 inflammasome stimulation is associated with the causative pathology of chronic kidney disease (CKD). Allopurinol (ALLO) represses xanthine oxidase (XOD) action and subsequently attenuates the generation of uric acid (UA) and reactive oxygen species (ROS), which are known to kindle the NLRP3 pathway. Hence, ALLO can impede the progression of CKD. Foresto-Neto et al., 2018 [19] probed whether suppression of XOD by ALLO attenuated NLRP3 stimulation and renal damage in the 5/6 renal extirpation (Nx) model. Adult male Munich–Wistar rats experienced Nx and were allocated into two arms, viz., Nx, administered vehicle only, and Nx + ALLO, Nx rats delivered ALLO, at a dose of 36 mg/kg/day in drinking water. Rats that underwent sham intervention were investigated as controls (C). Sixty days following surgery, Nx rats displayed notable albuminuria, creatinine retention, hypertension, as well as glomerulosclerosis, tubular injury, and cortical interstitial expansion/inflammation/fibrosis. Such alterations were tailgated by heightened XOD effects and UA renal concentrations related to boosted heme oxigenase-1 and attenuated superoxide dismutase-2 renal constituents. Both the NF-κB and NLRP3 signaling cascades were triggered in Nx. ALLO regularized both XOD effects and the measures of oxidative burden. ALLO also decreased hypertension and bolstered selective tubulointerstitial defense, decreasing urinary NGAL and cortical interstitial injury/inflammation. ALLO inhibited renal NLRP3 stimulation without meddling with the NF-κB signaling system. These results hint that the tubulointerstitial anti-inflammatory and antifibrotic actions of ALLO in the Nx model engage suppression of the NLRP3 pathway and bolster the belief that ALLO can arrest or impede the progression of CKD.

The therapeutic interventions in gout involve urate-decrementing therapy as well as acute and chronic anti-inflammatory agents. Allopurinol, a xanthine oxidase inhibitor, is the principal urate-lowering agent engaged for many years. Recent studies provide a hint that cardiovascular disease and lethality, chronic kidney affliction, prostate carcinoma, and manic manifestations are mitigated in patients suffering from gout who have been administered allopurinol. These findings uphold that allopurinol adds to a range of salutary actions beyond urate lowering. Multiple processes have been proposed to help explain conferment of such benefits, including build-up of adenosine and suppressive actions of allopurinol upon reactive oxygen species, tumor necrosis factor- α, nuclear factor kappa-light-chain-enhancer of activated B cells, as well as the NACHT, LRR, and PYD segments-containing protein 3 (NLRP3) inflammasome. Furthermore, allopurinol inhibits the activating actions of thioredoxin-interacting protein and directly negatively modulates the redox-active segment of thioredoxin, thereby decrementing NLRP3 activation. Allopurinol’s salutary analgesic and anti-inflammatory actions make it an attractive molecule for conducting supplementary studies and for the betterment of patient health [20].

### 3.2. New Synthetic Molecules

#### 3.2.1. Vinylsulfones (BAY11-7082)

Formerly described as an irreversible inhibitor of nuclear factor of kappa (NF-κB) pathway via IKKβ kinase activity abrogation, BAY11-7082 is a small molecule vinylsulfone with NLRP3 inflammasome repressive activity having IC50 of 12 μM [21]. By virtue of being a Michael acceptor, target protein inhibition is accomplished by causing alkylation of essential biological nucleophilic residues, such as glutathione and L-cysteines. By alkylating cysteine entities of the ATPase portion of NLRP3, BAY11-7082 suppresses the assembly of ASC pyroptosome and NLRP3 inflammasome signaling in NG5 cells as well as murine primary BMDMs. Moreover, BAY11-7082 was found to have greater specificity for NLRP3 over NLRP1, NLRC4 [21], and AIM2 [4] inflammasomes. In spite of its non-specific cysteine altering capability, BAY11-7082 was unable to directly suppress caspase-1. BAY11-7082 has also been purported to modulate several biomolecules like the ubiquitin system as well as tyrosine phosphatases [116]. Preclinical experimentation documents that these molecules have favorable pharmacokinetic properties, are non-mutagenic and well-tolerated, and possess optimal cell membrane permeability.

BAY11-7082 has been probed in a murine model of lupus nephritis, in which NF-κB and NLRP3 inflammasome stimulatory signaling pathways are unregulated [117]. Repression of proteinuria, renal dysfunction, cytokine secretion, and neutrophil migration has been demonstrated with BAY11-7082 in concert with a concomitant dip in mortality. In complicated maladies like systemic lupus erythematosus, drugs with manifold effects wield a crucial role. This characteristic of BAY11-7082 was also taken advantage of in a rodent model of neuropathic algesia employing lumbar disc herniation [118]. NF-κB was established as a pain modulator while concomitantly priming the NLRP3 inflammasome. Subsequent stimulation of the inflammasome maintained the inflammatory phenotype. In the neuropathic pain model, BAY11-7082 was administered at 5 mg/kg via ip injection thrice a week for 4 weeks [118]. This produced a statistically significant decrease in NF-κB, IL-1β, and IL-18 levels and a reduction in pain. Many other activities of BAY11-7082 also shored up the salutary outcome. Hence, this small molecule has a remarkable effect in vivo, and it would be exciting to find out if it can negotiate the blood–brain barrier in order for it to produce benefits in CNS disease models.

NLRP3 stimulation is evoked due to burn-induced acute lung injury [34]. At the cellular level, alveolar macrophages bolster NLRP3 expression and invigoration from burn serum, and this can be abrogated by BAY11-7082. This effect had been again demonstrated in vivo where IL-1β and IL-18 levels peaked at 24–48 h following damage [34]. BAY11-7082 has been administered at a dose 3 mg/kg by ip injection instantly following burn-evoked acute pulmonary damage. Roughly a two- to three-fold attenuation of NLRP3-associated inflammatory cytokines and proteins was attained with a concomitant decrease in myeloperoxidase. Histopathologic hallmarks of the injury, characterized as neutrophil intrusion, oedema, alveolar wall thickening and hemorrhage, were all relieved.

#### 3.2.2. Beta-Nitrostyrenes (MNS)

While screening a library of 160 molecules targeting kinases in murine BMDM cells, which were primed with LPS, 3,4-Methylenedioxy-β-nitrostyrene (MNS) was identified [24]. The power to result in a blockade at the NLRP3 inflammasome ATP-interaction site is understandable because libraries like these commonly contain molecules focusing on ATP-binding kinase domains. MNS, a documented Syk kinase suppressor, was spotted with an IC50 of 2 μM [22]. MNS ushered in abrogation of IL-1β, IL-18 secretion and active caspase-1 generation but not by changing mRNA expression amounts of inflammasome constituents. In a fashion akin to BAY11-7082, MNS behaves like a Michael acceptor; the role of the nitrovinyl side chain is integral for its biological action as a repressor of the NLRP3 signaling cascade. Alteration to the benzodioxole ring was managed but decreased the potency of the molecule. MNS activity focuses on the NLRP3 inflammasome effects on the leucine-rich repeat as well as nucleotide-binding oligomerization agglomerates simultaneously interacting with the ATP-binding site. With this reactive disposition, it is amazing that other inflammasomes, NLRC4, or AIM2 were not suppressed by MNS.

HMNS, a biotinylated probe and an active MNS analog, efficiently inhibited NLRP3 protein from cell lysate and could also abrogate recombinantly expressed NLRP3 [22]. This interaction was inhibited in the company of extra HMNS. On the other hand, by engaging biotin-HMNS, it was not possible to isolate NLRC4. Comprehensive NLRP3 and pyrin mutants or LRR segment mutants were segregated, engaging biotin-HMNS. Similarly, the NOD and LRR segments could be segregated, whereas this was not possible for the pyrin domain. Not many in vivo studies with MNS have been documented following its characterization as an NLRP3 regulator. A study conducted with MNS in a rodent wound healing model subsequent to burn injury facilitated healing [119]. Nitro-containing compounds and styrenes have not been observed to possess drug-likeness and find documentation in the scientific literature as toxicophores.

#### 3.2.3. Acrylate Derivatives (INF4E, INF39)

Cocco et al., 2014 [23] came up with the observation that Michael acceptor functionality was an intrinsic property of a majority of inflammasome inhibitors. They conjectured this permitted suppression of the cascade via interaction with cysteine moieties in caspase-1, NLRP3, or other pertinent proteins in the pathway. A library was hence fashioned about this electrophilic pharmacophore with the purpose of unravelling new irreversible NLRP3 inhibitors, which could be improvised for selectivity and negligible toxicity enhancements. As observed with other documented irreversible NLRP3 inhibitors, most of the new agents repressed caspase-1 and NLRP3 ATPase effects consistent with prediction. Of the 36 compounds, INF4E and its two close structural analogs were deemed to have the greatest potential for future testing [23].

INF4E and its congeners were not faultless; a level of cellular toxic damage was identified. INF4E is also irreversibly associated with human serum albumin giving rise to three covalent adducts, which have the potential to evoke idiosyncratic adverse reactions in vivo [120]. Therefore, this lead molecule was methodically altered [121] by extirpation or replacement of the alcohol component, hydrolytic cleavage of the ester to the carboxylic acid, and a few compounds also reduced the alkene, hence eliminating the probability of a covalent mode of effect. All compounds were prioritized via assays for Michael acceptor reactivity, cytotoxicity, NLRP3 activity, and pyroptosis in order to finally determine the upgraded molecule INF39. INF39 has been documented to possess a potency in the micromolar range; a 10 μM concentration attenuated IL-1β release by about 50% from LPS-primed murine BMDM. INF39 was deemed to be more specific than INF4E since no effect was exhibited on caspase-1. There was a 52% inhibition of NLRP3 ATPase action by INF39 at 100 μM concentration along with partial attenuation of NLRP3 inflammasome activation initiation. Expeditious biotransformation through INF39 ester hydrolysis to the carboxylate was identified in the course of permeation via rat intestine (ex vivo) and in the course of in vitro microsomal stability experiments. Both the acid and ester are operative as NLRP3 repressors, and none were toxic to the cells against THP-1 cell line (MTT assay) up to 100 μM concentration.

Owing to its high insolubility and lipophilicity, INF39 had been formulated as a suspension in olive oil intended for dosing by the oral route in vivo [121]. A rodent model of 2,4-dinitrobenzenesulfonic acid-evoked colitis was studied where INF39 was administered at 12.5, 25, and 50 mg/kg/day for 6 days starting at the initiation of colitis [121]. Salutary results were reported for body and spleen weight, colonic length, and observable injury, whereas decrements in concentrations of IL-1β, TNF-α, and myeloperoxidase have been observed. In spite of promising early results, it is challenging to verify the wider use of INF39 without subsequent studies. It is imperative to assess the pharmacokinetic profile and distribution parameters (either as the ester prodrug or the acid form) of INF39, simultaneously with a comprehensive inquiry of the toxicity profile. Substitute prodrugs or salt derivatives may facilitate the resolution of solubility problems and aid fitness of this compound in varied disease models in cases where direct delivery of the drug to its location of action is highly problematic.

As an augmentation of the acrylate containing covalent drug inquiry, combination analogs were generated and probed for their NLRP3 repressive effect [120]. Glyburide, a sulfonylurea agent for use in Type 2 diabetes, a weak NLRP3 active insulin secretagogue, and its forerunner sulfonamide, 16673-34-0, were added to the INF39 acrylate warhead. Both cross-bred entities maintained Michael acceptor reactivity, without any cytotoxicity at 100 μM to THP-1 cells and nonreactive with serum albumin. Attenuation of THP-1 pyroptotic cell lethality at 10 μM concentration of test compound was more efficacious for the 16673-34-0 conjugate at 46% compared with 17% for the glyburide crossbreed. While IL-1β secretion from wild-type mouse BMDM was roughly 50% at 20 μM concentration of the 16673-34-0 crossbreed, the effect was markedly blunted in macrophages carrying NLRP3-activating mutations usual of NLRP3-selective genetic disorders like Muckle–Wells. Attenuation of NLRP3 ATPase effect by the 16673-34-0 crossbreed was not of strong intensity with IC50 of 74 μM.

#### 3.2.4. Acylhydrazone (EMD638683)

EMD638683 has been documented to be an inhibitor of serum- and glucocorticoid-inducible kinase 1 and is known to inhibit NLRP3 inflammasome [24]. A high dose of EMD638683 (600 mg/kg/day in chow) given to mice attenuated cardiac inflammation and fibrosis evoked by angiotensin II. A comparison was made with MCC950 intervention (10 mg/kg ip), and comparable efficacies were documented. Subsequent administration of EMD638683 intervention demonstrated suppression of NLRP3 and IL-1β expression as measured by mRNA expression concomitantly with attenuation in cleaved caspase-1 and IL-1β in cardiac tissues of the exposed mice. Murine BMDM were employed to juxtapose the effect of EMD638683 to NLRP3 siRNA and also MCC950 (alone and also in combination) on attenuation of IL-1β levels, and results were comparable in all instances. EMD638683 has, thus far, only been tested as an experimental tool in animal models. EMD638683 is an acylhydrazone compound, and members of this chemical group are classified in medicinal chemistry as toxic molecular functionalities. In one study, EMD638683 evoked enhanced fluid uptake and urination simultaneously with a significant decrement in body weight [122]. Comprehensive toxicity charting is imperative in case this compound is to be developed further.

#### 3.2.5. Benzimidazoles (FC11a-2)

Fc11a-2 (a benzimidazole compound) suppressed the release of IL-1β and IL-18 from LPS-primed THP-1 cells activated with ATP exhibiting an IC50 of about 10 μM [25]. However, even at the highest tested concentration (30 μM), complete suppression of cytokine secretion was not achieved. Subsequent studies demonstrated that Fc11a-2 was unable to abrogate the priming stage but inhibited the splitting of pro-caspase-1 and so the proteolytic transformation of pro- IL-1β and pro-IL-18. In a dextran sodium sulfate (DSS)-evoked murine model of colitis, Fc11a-2 (10–30 mg/kg, administered by intragastric route) was found to dose-dependently improve the disorder with beneficial effects on body weight, colon length, histopathologic scoring, and myeloperoxidase activity [25]. Macrophage migration and active caspase-1 were also decreased concomitantly with a marked decrement in mRNA expression levels for IL-1β, IL-18, TNF-α, IL-17A, and IFN-γ. These outcomes provoked subsequent testing of analogs to comprehend the SAR. However, no analogs were markedly more potent in comparison with FC-11a-2, which at 10–30 μM demonstrated suppression of only 40% in cell-based assays mentioned earlier [26].

#### 3.2.6. Organoboron NLRP3 Inflammasome Inhibitors

Boron is quite a neglected element in medicinal chemistry and rarely employed in pharmaceutical compounds with only bortezomib (Velcade), crisaborole, and tavaborole in clinical use. An attractive group of NLRP3 inhibitors have been both documented [123] and patented [124], harnessing the boron semimetal scaffold 2-aminoethoxy diphenylborinate (2APB). Regrettably, in addition to suppressing NLRP3, 2APB also exhibits non-selective actions on cellular Ca^2+^ balance. Till recently, this parent scaffold was believed to disrupt cellular Ca^2+^ homeostasis via a multitude of processes, leading to NLRP3 inflammasome inhibition. However, Kastnelson et al. recently reported that NLRP3 inflammasome suppression by 2APB was unrelated to its effects on Ca^2+^ channels [125]. This work led investigators to an intensive search for analogous boron-based NLRP3 suppressive compounds lacking the cellular Ca^2+^ homeostatic modulatory effects. In the course of these experiments, the cyclic nature of 2APB was employed to scan through the zinc directory for agents with identical pharmacophore and shape; supplementary boron-containing agents were also pin-pointed via searches of SciFinder Scholar [123]. Two of the most potent early hits were BC7 (NLRP3 IC50 1.16 μM) and BC23 (NLRP3 IC50 2.29 μM). In the course of SAR reviews, the ring oxygen, boron, NH, and constituent CCl3 were all demonstrated as mandatory for compound effectiveness. The bisphenyl remained unchanged, but the rest two ring constituents could be altered to regulate activity. Entities NBC6, 18, and 24 had enhanced potency with interesting physicochemical attributes. The lead agent NBC6 possessed NLRP3 IC50 0.57 μM, which was markedly more potent in comparison to the original 2APB (NLRP3 IC50 67 μM). Thankfully, no effect was reported on cellular calcium equilibrium in in vitro cell-based estimations. NBC6 was well characterized as a selective repressor of NLRP3 inflammasome up to the highest concentration of 30 μM in murine BMDM or neutrophils. Both canonical and non-canonical inflammasome cascades could be abrogated to inhibit IL-1β release, but IL-1α secretion was unaffected. NLRC4 or AIM2 inflammasomes were unaffected by NBC6, exemplifying that the agent had an extent of selectivity across inflammasomes. Notably, NBC6 was investigated in washout cell-based estimations and was demonstrated to be an irreversible NLRP3 inflammasome suppressor.

A close analog of NBC6 called NBC13 (documented as easier to formulate but equipotent) was investigated in a murine model of peritonitis [123]. Both wild-type and NLRP3 knockout mice were employed, and MCC950 was investigated concomitantly as a comparator agent. NBC13 did indeed abrogate LPS-evoked IL-1β in vivo but not to the identical extent as MCC950. This could possibly be related to pharmacokinetic problems, but related information was not available in this context. However, this NBC series of boron-containing agents seems to be an exciting novel compound group destined to carve out a niche for future application in inflammatory afflictions.

#### 3.2.7. Sulfonylnitriles (OLT1177 (Dapansutrile))

Dapansutrile (OLT1177) is chemically a β-sulfonyl nitrile compound that has been demonstrated to suppress NLRP3-mediated IL-1β secretion from LPS stimulated PBMCs of cryopyrin-associated periodic syndrome (CAPS) patients and primary human neutrophils exemplifying the interception of IL-1β alteration in a classic NLRP3 gain-of-function disease [27,28]. Supplementary NLRP3-independent mechanisms of anti-inflammatory actions have been propounded for this molecule following testing in murine models. The entity was effective in vivo, attenuated the intensity of joint inflammation in a murine model of acute arthritis and retained myocardial function in mice subsequent to ischemia and reperfusion injury [126,127].

In Phase 1 clinical trial, OLT1177 demonstrated favorable pharmacokinetic parameters in healthy volunteers with an extended half-life and absence of hematological or organ toxicity at any of the administered doses and at plasma levels up to 310.9 mM [27]. A freshly concluded Phase 2 proof of concept research established that OLT1177 might have an adequate anti-inflammatory effect in patients afflicted with acute gout [128]. OLT1177 is presently under scrutiny in 3 clinical research programs, viz., acute gout flare, heart failure, and Schnitzler’s syndrome (EudraCT: 2016-000943-14; NCT03595371; NCT03534297). Against the backdrop of the involvement of NLRP3 inflammasome stimulation in multiple long-standing maladies like heart failure, neurodegenerative conditions, and metabolic syndrome, long-term research projects with OLT1177TM are imperative to establish the safety and effectiveness of this agent when given for extended periods of time [129].

#### 3.2.8. Benzoxathiole Derivatives (BOT-4-One)

BOT-4-one is a known covalent modifier (alkylating agent) with anticancer [130] and immunomodulatory actions [131]. Akin to various covalent modulators that repress NLRP3, this agent blunts its ATPase activity [29], providing a tip that interacts directly with NLRP3. Exposure of cells to BOT-4-one is also associated with increased ubiquitylation, but it is unclear whether BOT-4-one bolsters ubiquitylation or abrogates deubiquitylation. The compound also exhibited no effect on the AIM2 inflammasome and a not so intense inhibition of IL-1β after NLRC4 inflammasome stimulation. BOT-4-one abrogated IL-1β release and ASC oligomerization following NLRP3 inflammasome priming with no effect on acetylation of a-tubulin. Moreover, the agent failed to alter mitochondrial membrane potential consequent upon nigericin priming, another trigger for NLRP3 inflammasome assembly [30,31].

The anticancer properties of BOT-4-one are mediated through the blockade of janus kinase 3 (JAK3)/signal transducer and activator of transcription 3 (STAT3) signaling [130] and immunomodulatory action via suppression of IKKb [131]. Upon testing for NF-kB-unrelated effects, BOT4-one was found to act akin to other alkylating agents like Bay 11-7082 and MNS, decrementing IL-1β release following canonical and noncanonical NLRP3 inflammasome stimulation, but with enhanced potency [29].

In vivo, it was demonstrated that intraperitoneal administration of BOT-4-one (10 mg/kg) conspicuously decreased neutrophil intrusion and IL-1β levels in the peritoneal chamber following administration of MSU crystals. No notable actions were demonstrable on TNFα levels.

#### 3.2.9. Tryptophan Derivative/Metabolite (Tranilast((*N*-(3,4–dimethoxycinnamoyl)-Anthranilic Acid))

Tranilast is a tryptophan derivative that suppresses NLRP3 inflammasome polymerization and activation on account of its direct binding with its NACHT domain [4,32]. Tranilast (200 mg/kg) has been documented to increase body weight and survival and to produce demonstrable therapeutic benefits in murine models of NLRP3-induced gouty arthritis, MSU-induced peritonitis, and cryopyrin-associated periodic syndromes (CAPS). Moreover, tranilast attenuated caspase-1 autoactivation and IL-1β secretion from isolated synovial fluid mononuclear cells of arthritis patients [32]. Ever since its identification in 1982 as an anti-allergic agent that prevents histamine release from mast cells, tranilast use has been related to an increasing list of anti-inflammatory disorders (viz., bronchial asthma, atypical dermatitis, allergic conjunctivitis, keloids and hypertrophic scars). Tranilast inhibits TGF-β (major action), NFκB, and MAPK signaling pathways, and hence it may repress inflammation by inhibiting NLRP3 activation as well as by many other mechanisms. Moreover, tranilast has no modulatory actions on the upstream signaling events of the NLRP3 inflammasome, like NLRP3 and pro-IL-1β expression, ROS generation, K^+^ efflux, chloride efflux, and mitochondrial harm. Tranilast has exhibited inhibitory potential for homologous passive cutaneous anaphylaxis. The salutary effects of tranilast have also been demonstrated in a multitude of afflictions, like fibrosis, proliferative disorders, cancer, cardiovascular problems, autoimmune disorders, ocular diseases, diabetes, gout and renal diseases. Furthermore, some studies have reported that its administration has been associated with a low frequency of adverse effects, and it is usually well-tolerated by patients [132,133].

Hence, considering its clinical safety, tolerability, and efficacy profile in different disorders, tranilast is currently undergoing evaluation in phase 2 open-label clinical trial for efficacy and safety in CAPS patients (ClinicalTrials.gov Identifier: NCT03923140).

#### 3.2.10. Benzo[*d*]imidazol-2-One Compounds (HS203873, HS206461)

Liao et al., 2019 [134] spotted a novel pharmacophore that efficiently competes against ATP-binding of the NLRP3 protein, signifying that the ATP binding pockets of the other NLRPs could be alluring therapeutic targets as well. In this work, ATP Sepharose was employed in concert with a florescence-linked enzyme chemoproteomic strategy (FLECS) screen to determine potential competitive inhibitors of NLRP3. The work pinpointed a novel benzo[d]imidazol-2-one molecule which categorically targeted the ATP-binding and hydrolysis properties of the NLRP3 protein. HS203873, a benzo[d]imidazol-2-one compound, was determined to attenuate the ATP-binding, ATP hydrolysis, and oligomerization properties of NLRP3. HS203873 was the most compelling attenuator of ATP-provoked IL-1β secretion, to ~35% of the vehicle control. Moreover, compound HS206461 exhibited some inhibitory potential on ATP-induced IL-1β secretion [134].

### 3.3. Natural Products

#### 3.3.1. Beta-Hydroxybutyrate

In situations of multiple varieties of caloric constraints or energy insufficiencies, increased formation of β-Hydroxybutyrate (BHB), a ketone metabolite, has been documented to afford an alternative fuel source. BHB also serves in cell signaling pathways [135]. Under these conditions, an anti-inflammatory action associated with the innate immune response has been reported recently [33]. Youm et al. [33] demonstrated that BHB efficiently repressed K^+^ outflow (a well-documented NLRP3 inflammasome activator) while also impeding ASC polymerization and speck generation. BHB was determined to be efficacious in suppressing stimulation of the NLRP3 inflammasome with an IC50 ~1 mM, which is a physiologically pertinent concentration [33].

A murine model of the NLRP3-selective human disease familial cold autoinflammatory syndrome (NLRP3 (L351P) Cre^+^) correlated ketogenic (to increment BHB) with chow diet [33]. In this disease model, NLRP3 is stimulated, evoking disproportionate neutrophilia [136]. It was established in these experiments that neutrophilia and hyperglycemia were circumvented by the administration of a ketogenic diet. Hence, heightened BHB concentrations have been reported to be favorable in the setting of inflammatory disorders. In order to accomplish the dosing of BHB in vivo, a nanolipogel complex had been employed as this formulation protracts the half-life of BHB. BHB has been noted to produce beneficial outcomes in published animal (mice and rat) models of gout, a disorder kickstarted by monosodium urate (MSU) crystals inducing macrophage stimulation with consequent inflammasome-mediated, neutrophil intrusion culminating in excruciating algesia and swelling. In the murine model, intraperitoneal injection of monosodium urate crystals was given to evoke the inflammatory reaction; administration of nanolipogel formulation of BHB (125 mg/kg ip) decreased heightened circulating IL-1β levels and precluded intrusion of neutrophils into the peritoneum [33]. A subsequent rat model of MSU crystal-induced gout was tested. Outbred Sprague–Dawley rats were fed with a ketogenic or chow diet and subsequently injected with MSU crystals in the knee. Notable attenuation of inflammatory response and tissue injury was evident in the rats fed the ketogenic diet. There was no notable elevation of circulating IL-1β concentrations in ketogenic diet-fed rats. BHB ameliorated the disease process in an equieffective manner in both adult and elderly mice; furthermore, there was no aggravation of *Staphylococcus aureus* infection owing to BHB, implying that immune response to pathogens was not tampered with.

BHB has been tested in an experimental rodent model of Parkinson’s disease (PD). PD was produced in rats by LPS exposure to the right substantia nigra pars compacta. BHB was delivered round the clock through subcutaneous osmotic pump at three doses (0.4, 0.8, 1.6 mmol/kg/day = 41.6, 83, 166 mg/kg/day) starting from 3 days before the LPS exposure till 3 weeks post-treatment. There appeared to be a dose-dependent motor function improvement assessed by amphetamine-induced rotational behavior. However, total symptom amelioration could not be achieved. A dose-dependent increase in concentrations of dopamine and dopamine metabolite (DOPAC) was noted. The beneficial action of BHB on dopaminergic neurons was evident from Western blot and immunohistochemistry analyses [137].

Following identification of elevated IL-1β, IL-6, and TNF-α levels in the pathophysiology of major depressive disorder, the interaction of BHB with NLRP3 inflammasome was probed in a relevant murine model of this disorder [138]. IL-1β was associated with antineurogenic and anhedonic behavior, and symptom alleviation occurred upon administration of IL-1β receptor antagonist (IL-1Ra) or by knocking out the IL-1β receptor (IL-1RI) [139]. BHB exposure in an immobilization stress model in mice led to a minute but statistically significant attenuation in IL-1β levels. On the other hand, no alteration in IL-1β level was reported upon BHB exposure in a chronic unpredictable stress model [138]. Further investigations are warranted for dissecting the actual beneficial mechanisms.

#### 3.3.2. Isothiocyanate Compounds Derived from Glucosinolates

Glucosinolates are a naturally occurring group of about 130 biologically effective compounds found in all plants belonging to the mustard family. Multiple plants from this family are referred to as “superfoods” owing to their useful biological actions offered by the glucosinolate system [140]. Sulforaphane, obtained from the glucosinolate glucoraphanin, was tested at the National Institute for Health in Bethesda for suppressing inflammasome activation [140]. Multiple inflammasomes were suppressed, viz., NLRP1b, NLRP3, NAIP5/NLRC4, and AIM2 post-priming. Sulforaphane was reported not to be a direct caspase-1inhibitor, in spite of having a cysteine residue at the active site. Though multiple cascades were probed to determine the mechanism of suppression, such as determination of the heat-shock effect, action on Nrf2, and tubulin alteration, these did not provide a conclusive result. More research is imperative. Sulforaphane (25 mg/kg, ip at 0 and 4 h) was also probed in an acute murine (C57BL/6) model of monosodium urate crystal-evoked gout [140]. In this model, the NLRP3 inflammasome is normally stimulated due to the crystalline deposits leading to the liberation of IL-1β and migration of inflammatory cells. Peritoneal lavage fluid obtained from sulforaphane MSU-exposed mice contained markedly attenuated levels of IL-1β as well as recruited cell count in comparison to the vehicle MSU-treated control animals. Parent glucosinolates are known to be present in Cruciferae (especially the Brassica vegetables). Sulforaphane has reportedly undergone testing in NLRP3 inflammasome-related disorders like osteoarthritis, COPD, AMD, diabetes, and cardiovascular disease [141].

#### 3.3.3. Sesquiterpene Lactones (Parthenolide, Arglabin, Artemisinin)

Parthenolide is a naturally occurring sesquiterpene lactone of germacranolide class in the Feverfew plant (*Tanacetum parthenium*) [142]. The maximum concentration of parthenolide occurs in the flowers and fruit. This natural product is a covalently reactive compound and has been probed for its effectiveness against a multitude of various diseases such as hypoadiponectinemia, infection (bacterial or parasitic), neoplastic disorders, and inflammatory disorders [142]. Feverfew has been tested for its therapeutic salutary actions in migraines in clinical trials [143].

Parthenolide can repress NLRP3 inflammasome-activated cytokine liberation with an IC50 of 2.6 μM in the THP-1 cell line by various mechanisms [144]. Parthenolide impedes the NF-κB signaling pathway, which modulates transcription of NLRP3 inflammasome constituents during the cell-priming stage [34]. Toll-like receptor activation with LPS can induce stimulation of the NF-κB pathway. NLRP3 inflammasome formation can then be induced by a second alert, like ATP. In 2010, parthenolide was determined to also suppress the NLRP3 inflammasome formation step [34]. In LPS-primed bone marrow macrophages treated with parthenolide, no inflammasome formation could take place. The study by Juliana et al. showed that parthenolide’s suppressive effects were not confined to its actions on the NF-κB pathway. It suppressed caspase-1 stimulation in response to NLRP1, NLRC4, and NLRP3 activation by alkylating many cysteine moieties of caspase-1. Parthenolide targets ATPase actions of NLRP3 protein directly, possibly via cysteine alteration. In view of its poor solubility and bioavailability, its water-soluble analogs are being evaluated currently [145,146].

Arglabin is found naturally in *Artemisia glabella* and belongs to the guaianolide class. *Artemisia glabella* is a type of wormwood found only in Kazakhstan [147]. Arglabin suppressed the NLRP3 inflammasome in LPS-stimulated murine BMDM treated with cholesterol crystals, employing IL-1β and IL-18 liberation as outcome measures [147]. No inhibitory effects (up to 50 nM) were noted upon NLRP1, AIM2 or NLRC4 inflammasome when activated by their corresponding activating stimuli [147]; probing higher concentrations of arglabin in experimental models is imperative before declaring true selectivity.

Cholesterol crystals are known to commonly trigger inflammasome activation in atherosclerosis. Arglabin has been tested for its efficacy in in vivo ApoE2.Ki mice expressing human ApoE2 (2/2) following initial reports of its cytokine inhibitory effect in response to cholesterol crystals [148]. Upon administration of an atherogenic diet, these mice are predisposed to generate atherosclerotic plaques. A twice-daily low dose of arglabin (2.5 ng/g ip) for 13 weeks efficaciously attenuated about 50% plasma levels of IL-1β. A 59% attenuation of total cholesterol, 42% reduction in triglycerides, and 44% decrement in autoantibodies towards oxLDL were noted when compared to vehicle-treated mice. Furthermore, macrophage phenotype switching from pro-inflammatory M1 to the anti-inflammatory M2 was demonstrated in spleen and arterial tissues. Both aortic sinus and whole aorta en face in arglabin administered mice were attenuated to identical levels as detected in ApoE2. Ki/NLRP3−/− mice were kept on an atherogenic diet. Disparities were not observed in LDL receptor generation, hepatic steatosis, or cholesterol biosynthesis. In a supplementary study in an identical murine model, arglabin reduced plasma glucose concentrations (20% reduction) and insulin levels (50%) [147]. Further research is imperative to bolster the positive in vivo results and to focus on the potency and selectivity of this compound.

Artemisinin (sesquiterpene lactone) is an antimalarial drug occurring naturally in *Artemisia annua* (sweet wormwood). There are many analogs of this compound in therapeutic use. This medication is usually well-tolerated and effective in multiple disease models [149], such as inflammatory scenarios like post-infarct myocardial remodeling, mouse EAE model of multiple sclerosis, and lupus nephritis. One observed mechanism of artemisinin action is through repression of the NF-κB signaling cascade to regulate the immunological effect, as noted in human astrocytoma T67 cells [150] and also in microglia [151]. This effect of artemisinin is normally thought to be responsible for NLRP3 inhibitory effects in a transgenic murine (APPswe/PS1dE9) model of Alzheimer’s Disease [152]. Once-daily treatment with artemisinin (dosed at 40 mg/kg ip) attenuated amyloid plaques in the cortex by 48% and in the hippocampus to the tune of 61%. Amyloid-β movement across the blood-brain barrier was maintained. However, amyloid precursor peptide cleavage was inhibited due to enzyme β-secretase (BACE1) inhibition [152]. Artemisinin profoundly affected BACE1 expression by suppressing NF-κB expression and translocation to the nucleus. The NLRP3 priming phase is also associated with NF-κB nuclear translocation, and understandably NLRP3 signaling pathways were inhibited concomitantly with a consequent decrease in cleaved caspase-1 and IL-1β generation.

The NLRP3 regulatory effects of artemisinin were also demonstrated in a murine model of burn sepsis [153]. Dry eye disease (DED) is a multifactorial malady of the tears and ocular surface featured by a display of dryness and irritation. Although the disease pathology has not been completely dissected, it is obvious that inflammation has a conspicuous role in the progression and worsening of DED. β-aminoarteether maleate (SM934) is a water-soluble artemisinin analog with anti-inflammatory and immunosuppressive effects. In this research, the group demonstrated that the scopolamine hydrobromide (SCOP)-evoked rodent model and benzalkonium chloride (BAC)-activated rat model were truly representative models to corroborate the therapeutic utility of SM934 for DED. It was demonstrated that topical administration of SM934 (0.1%, 0.5%) notably enhanced tear secretion, retained the number of conjunctival goblet cells, attenuated corneal injury, and decreased the levels of inflammatory cytokines (TNF-α, IL-6, IL-10, or IL-1β) in conjunctiva in SCOP-kindled and BAC-evoked DED models. Also, SM934 treatment attenuated the aggregation of TLR4- expressing macrophages in conjunctiva, and decreased the generation of inflammasome constituents, i.e., myeloid differentiation factor88 (MyD88), NOD-like receptor protein 3 (NLRP3), apoptosis-associated speck-like protein containing CARD (ASC), and cleaved caspase-1. In LPS-treated RAW 264.7 cells, it was established that preadministration with SM934 (10 μM) thwarted the upregulation of TLR4 and downstream NF-κB/NLRP3 signaling protein cascade. Taken together, artemisinin derivative SM934 produced therapeutic usefulness in DED by concomitantly preserving the structural rectitude of ocular surface and obviating corneal and conjunctival inflammation, hinting towards a subsequent application of SM934 in ophthalmic therapy, specifically for DED [154].

The role of artemisinin in myocardial ischemia/reperfusion (I/R) injury and the involvement of NLRP3 inflammasome were tried in an animal model. Artemisinin was administered for 14 consecutive days intragastrically before I/R injury. Cardiac function was assessed by echocardiography. The infarct area was observed through HE and TTC staining. Apoptosis and autophagy were assessed by TUNEL and Western blotting. The artemisinin-treated myocardial I/R rats demonstrated less severe myocardial I/R injury (smaller infarct size and lower CK-MB, LDH), significant inhibition of cardiac autophagy (decreased LC3II/I and increased p62), improved mitochondrial electron transport chain activity, concomitant with decreased activation of NLRP3 inflammasome (decreased NLRP3, ASC, cleaved caspase-1, IL-1β). In conclusion, our findings further confirmed that activation of the NLRP3 inflammasome pathway is involved in myocardial I/R injury, whereas artemisinin preconditioning could effectively protect against myocardial I/R injury through suppression of NLRP3 inflammasome activation. Therefore, the NLRP3 inflammasome might serve as a promising therapeutic target providing new mechanisms for understanding the effect of artemisinin during the evolution of myocardial infarction [155].

Artemisinin has been demonstrated to down-regulate the NF-κB/NLRP3 signaling cascade, ameliorating renal tubulointerstitial inflammation and fibrosis in 5/6-nephrectomized rats [156,157,158]. The artemisinin type of molecules is expected to occupy a wider arena of therapeutic application eventually.

#### 3.3.4. Flavonoids

Flavones are generally distributed across the human diet and may bestow health utilities of fruit and vegetable intake. Hence, this chemical group has mustered profound interest. Following intake, usual peak plasma flavone levels fluctuate, whereas isoflavones have a proclivity towards favorable bioavailability [159]. Flavones are poorly absorbed, extensively metabolized and readily excreted, leading to low bioavailability [159]. Additionally, there is a multitude of effects upon cell signaling. Of late, there has been an intensification of research on inflammasome regulation by flavones in vitro and in vivo. Past documentation of the anti-inflammatory efficacy of flavonoids zeroed in on the C2–C3 alkene, whereas the location of the hydroxyl groups is critical to the anti-inflammatory efficacy of flavonoids.

The flavonoid liquiritigenin, bearing close resemblance to chalcone isoliquiritigenin, and a saponin, glycyrrhizin, were extracted from *Glycyrrhiza uralensis* of the licorice plant group. Both isoliquiritigenin and glycyrrhizin repressed TLR4/MD-2 complex and IKK, precluding NF-κB stimulation and hence inflammasome arousal. Isoliquiritigenin showed greater potency in comparison to glycyrrhizin and displayed enhanced selectivity over AIM2 inflammasome. Both compounds repress priming and activation phases of NLRP3 inflammasome genesis. Using murine BMDM with inflammasome arousal (LPS priming with consequent ATP, MSU or nigericin trigger), NLRP3-stimulated IL-1β release was decreased by about 50%, as correlated with vehicle control, by 1–10 μM isoliquiritigenin, whereas 1 mM glycyrrhizin was needed to demonstrate an identical effect. Parthenolide was incorporated as a control in these assays, demonstrating almost identical activity to isoliquiritigenin and the closely similar flavonoid liquiritigenin. However, glycosylated forms of either isoliquiritigenin or liquiritigenin (liquirtin, isoliquirtin, liquirtin etoposide, and isoliquirtin etoposide) also occurring in *G. uralensis*, were devoid of any such activity. This correlates with documentation that deglycosylated flavones led to an enhancement of anti-inflammatory effects [160]. Stimulation of regulatory T cells in vitro and in vivo by isoliquiritigenin have been demonstrated in some studies [161]. In adipose tissues, inflammation was inhibited both in an inflammasome-dependent and an inflammasome-independent manner [162]. Therefore, the actions of flavones in regulating inflammation are varied and not only the outcome of inflammasome activation.

The bioflavonoid apigenin, which naturally occurs in fruit, vegetables, and herbs like chamomile, is structurally analogous to liquiritigenin. Apigenin has been demonstrated to suppress IL-1β secretion and NLRP3 activation and priming from THP-1 cells and J774A.1 cells [163]. There are indications of a probable relation among NLRP3 activation, stress, and major depressive disorder [75]. Apigenin was probed in vivo to calibrate its efficacy in a rodent model of chronic unpredictable mild stress [164]. An intragastric dose of 20 mg/kg of apigenin regularized sucrose usage, symbolic of antidepressant action, ameliorated behavioral correlates and decreased microglial stimulation. There was also a notable attenuation in levels of oxidative stress, IL-1β, IL-18, and production of NLRP3, caspase-1, and ASC proteins almost identical to that in the vehicle-only group.

In a rat model of intracerebral hemorrhage, isoliquiritigenin was probed with a favorable outcome, and various studies were subsequently undertaken to decipher its mechanism of action [165]. Anti-inflammatory interventions in intracerebral hemorrhage offer crucial promise against the backdrop of lack of any meaningful agents for this debilitating disorder. Prevention of brain damage is noted in NLRP3 knockout or inhibition, and the Nrf2 anti-oxidant signaling cascade obviates liberation of reactive oxygen species (ROS), a documented trigger for NLRP3 inflammasome activation. The rodent model of collagenase type IV-evoked intracerebral hemorrhage was executed employing three experiments [165]. The first experiment employed five groups with 36 rats in each group: sham, vehicle only, and isoliquiritigenin 10, 20, and 40 mg/kg. The 10 mg/kg group failed to exhibit a notable salutary effect, but 20 and 40 mg/kg groups were identically beneficial. Improvements were noted in behavioral inadequacies (motor, sensory, balance, and reflex) and histological activities, whereas hematoma magnitude, brain swelling, and BBB penetrability were attenuated. The second experiment was conducted using four groups of 30 rats in order to probe the mechanisms of therapeutic actions: sham, intracerebral hemorrhage only, vehicle administered, and isoliquiritigenin 20 mg/kg exposed. Experiments established that isoliquiritigenin stimulated expression and nuclear translocation of Nrf2. Isoliquiritigenin induced alkylation of reactive cysteine moieties causing stress sensing in the Nrf2 partner protein kelch-like ECH-associated protein 1 (Keap1) was hypothesized as the mechanism [165]. Isoliquiritigenin inhibited both the NF-κB pathway and NLRP3 inflammasome proteins (viz., NLRP3, ASC, pro-caspase-1, pro-IL-1β, and pro-IL-18) [165]. Due to the inactivation of the inflammasome signaling pathway, active IL-1β and IL-18 secretion was notably blocked. The third experiment employed siRNA for Nrf2 as well as isoliquiritigenin administration concomitantly. The Nrf2 siRNA group was markedly inactivated with aggravation of all previously named brain damage and inflammatory markers. Mitigation of injury was noted in the isoliquiritigenin treatment arm [165]. A thorough dissection of isoliquiritigenin’s anti-inflammatory therapeutic potential in this CNS affliction was possible in this scenario with certain outlined limitations.

In type 2 diabetes murine model, where C57BL/6 mice were exposed to a high-fat diet for a 20-week duration, isoliquiritigenin (0.5% *wt*/*wt* in chow) produced a salutary effect in the context of adipose tissue inflammation [166]. Cholesterol, triglycerides, leptin, and insulin levels in the serum were decreased, and a correction in insulin sensitivity was noted. Despite the fact that isoliquiritigenin-exposed mice ate up slightly more food, they attained less weight, and hepatic steatosis was mitigated. In the context of adipose tissue inflammation, there were undoubtedly reduced crown-like structures found around adipocytes, suggesting an attenuation in the number of inflammatory cells. In addition, there was also a decrement in the generation of inflammatory TNF-α, IL-6, and MCP-1 genes and an accretion in adiponectin levels. IL-1β and caspase-1 generation were noticeably attenuated at the 4- and 20-week time points accruing out of inflammasome modulation. Isoliquiritigenin produced undoubtedly salutary actions in this murine obesity model; however, not all of these effects were attributable to NLRP3 inflammasome suppression. Nevertheless, in an identical overnutrition animal model of NASH, MCC950-evoked NLRP3 inflammasome suppression failed to mitigate hepatic fatty changes and was without response to the metabolic consequences of the affliction [167].

Wogonoside, extracted from the blooming plant *Scutellaria baicalensis Georgi* (Chinese skullcap), prevents IL-1β liberation from LPS-exposed THP-1 cells evoked with ATP with an IC50 around 50 μM [168]. NF-κB nuclear translocation and DNA association was repressed in concert with pro-IL-1β and NLRP3 expression. Also, active caspase-1 was repressed in concert with attenuation of IL-1β secretion. Wogonoside (12.5, 25, and 50 mg/kg, administered by intragastric route) inhibited dextran sodium sulfate (DSS)-induced colitis in a dose-dependent fashion [168]. Wogonoside in similar doses was also tested in a rodent spinal cord injury model and determined to mitigate attending inflammation due to inhibition of NF-κB and NLRP3 inflammasome activation [169]. Wogonoside (10, 20, and 40 mg/kg) enhanced survival of BALB/c mice in a model of lipopolysaccharide (LPS) and d-galactosamine-evoked hepatic damage in a dose-dependent fashion by stimulating Nrf2 and inhibiting NLRP3 activation in a manner akin to other flavonoids [170].

Casticin (vitexicarpin) attenuated LPS-evoked acute lung injury; in a fashion akin to other flavonoids, the process was credited to inhibition of NF-κB and attenuation of NLRP3 inflammasome activation [171]. Rutin, a disaccharide quercetin derivative, exhibited the capability to regulate NLRP3 inflammasome in three models of inflammatory disease, viz., pancreatic inflammation, spinal cord damage, and endothelial dysfunction [172,173,174]. Quercetin, luteolin, and epigallocatechin gallate also regulated NLRP3 inflammasome in vivo [175,176,177,178]. Epigallocatechin gallate, in addition, has been noted to inhibit NLRP1 inflammasome in the melanoma growth model [179].

The anti-inflammatory actions of isorhamnetin and hyperoside (the 3-O-galactoside of quercetin) sourced from water dropwort (*Oenanthe javanica*) were examined engaging LPS-primed murine BMDM against inflammasome provoking agents for NLRP3, NLRC4, and AIM2 [180]. Isorhamnetin decreased IL-1β, IL-18, and cleaved caspase-1 levels associated with exposure to NLRP3 (ATP, nigericin, alum) and AIM2 (dsDNA) provoking agents, but these were heightened for NLRC4 provoking agent (flagellin). Pro-IL-1β, TNF-α, IL -6, and NLRP3 production, as a reaction to LPS, was abrogated by isorhamnetin. On the other hand, hyperoside failed to alter the levels of inflammasome proteins; this molecule suppressed the stimulation of NLRC4 inflammasome as well as AIM2 without affecting NLRP3 inflammasome [180]. Hence, there is a probability that many glycosylated flavones may have activity against AIM2 and NLRC4.

The efficacy of isoliquiritigenin and its near-similar derivatives in a multitude of disease models obviously resulted from multimodal mechanisms. These molecules were principally examined for actions on NLRP3 inflammasome, and substantial work remains to be done to note the actions on other inflammasome signaling pathways. Extensive biotransformation of the flavones [159] has not been considered, and it may be possible that a few actions observed may be due to these metabolites. This would have special relevance in the context of the liver and intestines, where extensive bioprocessing of flavonoids is known to occur. It is imperative that cellular and in vivo data interpretation need to be cautiously considered in the backdrop of bioavailability and probable in vivo levels [181]. Against the background of global human intake of flavonoid natural compounds, this group of molecules is obviously of special relevance.

#### 3.3.5. Quinones

Thymoquinone, which is sourced from black cumin seed (*Nigella sativa*), has been probed as an antineoplastic plant-derived chemical compound with diverse modes of action and related anti-inflammatory and immunomodulatory properties [182]. Thymoquinone has also been tested in vivo for NLRP3 associated actions in a murine model of cancer cell shift. The tail was employed to inject B16F10 cells, and pulmonary tumor lump production was employed as an outcome measure. Thymoquinone dose-dependently attenuated nodules from 15 to 1 in this model [183].

Thymoquinone has been probed for its NLRP3 related actions on pancreatitis [183]. Upon thymoquinone exposure (100 mg/kg p.o. for 2 months) to a murine model of pancreatic inflammation, there was a decrement in IL-1β, IL-18, and TNF-α mRNA levels (oxidative stress markers). Outcome parameters such as interstitial oedema inflammation and inflammatory cell infiltration, parenchymal cell necrosis, and hemorrhage showed a salutary effect of thymoquinone in experimental pancreatitis.

#### 3.3.6. Stilbenoids and Close Analogs (Cinnamic acid, Curcumin, Resveratrol, Rhaponticin, and Salidroside)

Cinnamic acid is typically sourced from cinnamon and possesses a Michael acceptor functionality usually endowed in many synthetic NLRP3 inhibitor agents (such as BAY11- 7082, MNS, acrylate derivatives). It would be fair to speculate that cinnamic acid could alter the NLRP3 inflammasome pathway proteins in a covalent fashion. However, direct proof of this is lacking. In an LPS-evoked endotoxin-poisoned murine model, cinnamic acid was probed for its therapeutic effectiveness [184]. A significant attenuation of neutrophilic infiltration was noted, as well as decrement of the pro-inflammatory cytokines IL-1β, IL-18, and TNF-α in serum. NLRP3, caspase-1, IL-1β mRNA, protein of NLRP3, and cleaved caspase-1 were determined to be attenuated as well. These data are suggestive that cinnamic acid can repress NLRP3 activation in pro-inflammatory situations.

In a rat model of acute hepatitis evoked by d-Galactosamine coupled with gamma irradiation (d-Gal/radiation), cinnamic acid nanoparticles (CANPs) guarded the hepatocytes from injury by a combination of its anti-inflammatory and antioxidant effects and by regulating oxidation cellular pathways leading to the acute severity of hepatitis. Upon oral delivery of CANPs, the severity of acute hepatitis and the serum enzyme levels of ALT, AST, and ALP were attenuated. In addition, the hepatic tissue levels of malondialdehyde (MDA) and nitric oxide (NO) have been significantly reduced. The total antioxidant activity (TAO) exhaustion was rapidly restored. Moreover, the attenuation of hepatic damage accruing out of pretreatment with CANPs was associated with significant repression in the concentrations of hepatic proinflammatory cytokines (like TNF-α, IL-1β, and IL-18), NF-κB, NLRP3, caspase-1, and proapoptotic protein BAX, whereas anti-apoptotic protein Bcl-2 level was markedly raised when correlated with d-Gal/radiation-induced acute hepatitis (AH) group. In this study, CANPs downregulated d-Gal/radiation-evoked IL-1β, IL-18, and ASK1 mRNA gene expression levels and TLR4 and MyD88 protein expression in liver tissue. Moreover, CANPs effectively suppressed apoptosis. Hence, cinnamic acid nanoparticles are endowed with the medicinal capability to safeguard the liver from acute hepatitis [185].

The same research group determined in another study that following oral dosing of CANPs, there was attenuation in the intensity of acute pancreatitis as well as attenuation in the serum levels of amylase and lipase. Additionally, there was a notable decrement in pancreatic tissue malondialdehyde (MDA) levels. The exhaustion of glutathione was markedly replenished. The injury and apoptosis of pancreatic tissues were markedly reduced by the attenuation of caspase-3 concentrations. Furthermore, the mitigation of pancreatic oxidative injury by CANPs was associated with a down-regulation of the NLRP3, NF-κB, and ASK1/MAPK signaling pathways. In summary, the study results showed that CANPs could safeguard the pancreatic acinar cells from inflammatory insult via their antioxidant, as well as anti-inflammatory properties and also by regulation of the injurious redox-sensitive signal transduction pathways aggravating acute pancreatitis. Hence, cinnamic acid nanoparticles have salutary effects for managing acute pancreatitis [186].

A trendy superfood, curcumin (a major polyphenolic bioactive constituent of turmeric) has seen an upswing in its use. Akin to a plethora of traditional natural products, curcumin has been researched in various disease models and has been touted to possess multiple modes of salutary actions. In a murine osteoarthritis model, where destabilization of the medial meniscus (DMM) surgery was employed, some degree of in vivo modulation of inflammasome was achieved by administration of curcumin [187]. Curcumin (administered at a dose of 50 μM, ip) impeded disease advancement and decreased IL-1β, IFN-γ, IL-17A, IL-18, TNF-α, and VCAM1 mRNA expression levels. Curcumin has been documented to have salutary effects in various murine models with scrutiny of NLRP3-driven associations like high-fat diet (HFD)-induced insulin resistance and MSU-induced peritoneal inflammation [188], chronic kidney disease [189], potassium oxonate-induced hyperuricemia and kidney inflammation [190], LPS-evoked septic shock [191], diabetic nephropathy [192], lupus [193], and dextran sulfate sodium (DSS)-induced colitis [194]. In a Sprague–Dawley rat model of kainic acid (KA)-induced epilepsy, curcumin suppressed KA-evoked epileptic syndromes via inhibition of NLRP3 inflammasome activation, opening up a potentially salutary effect in epilepsy [195]. In a Wistar rat model of MSU-evoked gouty arthritis, curcumin produced salutary effects by inhibiting NLRP3 inflammasome suppression via repression of NF-κB signaling both in vitro and in vivo [196].

Resveratrol is a stilbenoid that is usually sourced from the skin of grapes and other berries. This polyphenolic molecule has been tested in a multitude of studies exemplifying its bioactive potential in many disease models. Though resveratrol is efficiently absorbed (~70%), it, unfortunately, possesses undesirable oral bioavailability (about 0.5%), owing to extensive biotransformation [197]. In 2013, while probing radiation injury biochemical pathways, it was established that resveratrol could modulate the NLRP3 inflammasome [198]. Resveratrol triggered Sirt1 deacetylase inhibiting its capability in transactivation of NF-κB and consequent action on NLRP3 transcription [198]. Similar actions were also noted in microglia obtained from mice [199]. Further research reported that resveratrol repressed NLRP3 inflammasome assembly as well as priming. However, resveratrol is non-selective in its actions because it also regulates NLRP1 and NLRC4 inflammasomes [200]. Furthermore, the levels of the autophagy biomarker LC3B-11 was enhanced by resveratrol treatment; the autophagy pathway promotes degeneration of NLRP3 and pro-IL-1β, consequently repressing NLRP3 inflammasome activity [200]. NLRP3 activity status has been examined in in vivo murine renal inflammation models [200], LPS-evoked acute lung injury [201], adipose tissue inflammation [202], and sepsis-associated encephalopathy [199,200], amongst many others. The effectiveness of resveratrol in human aberrations has also been probed; in fact, clinical trials numbering about 130 have been recorded in clinicaltrials.gov, with 17 of those undergoing phase III.

Rhaponticin is a glycosylated stilbenoid sourced from rhubarb rhizomes. The aglycone portion is very similar to resveratrol and has an almost identical reported effect as a Sirt1 stimulator [203]. No testing has yet been reported with this compound on mitochondrial protection or autophagy. In a recent study, rhaponticin (dosed at 20–100 mg/kg, p.o.) was salutary in a dextran sodium sulfate (DSS)-evoked murine model of colitis [203]. Salidroside is a glycosylated form of tyrosol present in Rhodiola rosea (golden root) and is especially touted to be an antidepressant and anxiolytic. This agent has been tested and found to have salutary effects in diabetic nephropathy [204] and ventilation-induced pulmonary damage [205]. Like resveratrol and rhapontin, salidroside is a Sirt1 activator inhibiting transactivation of NF-κB and consequent NLRP3 activation [205]. Further research is imperative to bolster the in vivo data and to determine potency and selectivity of this compound.

#### 3.3.7. Steroids

When administered in a mouse model of cuprizone-induced demyelination, prednisone (at a dose of 10 mg/kg, p.o.), a potent anti-inflammatory steroid, produced NLRP3 modulatory actions [206]. Prednisone efficiently attenuated microglial and astrocyte activation as well as NLRP3, IL-1β and active caspase-1 protein levels. In order to back the in vivo data, settle potency and selectivity issues, more focused research is imperative.

#### 3.3.8. Pentacyclic Natural Products

Senegenin (tenuigenin) is sourced from the traditional Chinese medicinal herb *Polygala tenuifolia*, which has been touted to produce antidepressant effects, which were established in a chronic unpredictable mild stress mouse model. Effects were determined to be related to NLRP3 repression [207]. NLRP3-activated IL-1β secretion was abolished via NF-κB pathway inhibition as evidenced by hippocampal phosphorylation levels of NF-κB through Western blot and immunohistochemical techniques. In order to back these initial findings, more focused research is necessary.

Celastrol, which is present in the roots of *Tripterygium wilfordii* (thunder god vine), has been probed as an anti-inflammatory extract in clinical trials for rheumatoid arthritis in 2009 with some success [208]. Pyroptotic cell lethality is symbolic of NLRP3 stimulation and was attenuated by celastrol when assessed by LDH release assay. Celastrol repressed ROS through its well-established anti-oxidant effects and also blunted NF-κB stimulation [209]. A later study also established celastrol as an NLRP3 inhibitor and dished out greater detail [210]. Celastrol facilitated autophagy (known to repress NLRP3 inflammasome activation) and obviated ASC oligomerization as well as generation of the NLRP3 complex. Encouraging effects were noted in in vivo murine models of LPS-evoked septic shock where celastrol was dosed at 1 mg/kg ip, as well as in dextran sodium sulfate-evoked colitis. More investigations are warranted in this area.

#### 3.3.9. Alkaloids

Sinomenine (cocculine) is an alkaloid (with structural similarity to morphine) sequestered from *Sinomenium acutum* and conventionally employed to mitigate rheumatic, arthritic, and neuralgic maladies. In a murine model of ischemic stroke, sinomenine (10–20 mg/kg, ip) provided salutary effect by attenuating expression of NLRP3 and ASC, decrementing cleaved caspase-1 and IL-1β levels, as well as IL-6, IL-18, and TNF-α concentrations [211]. Sinomenine (30 mg/kg) has also been reported to be effective in a murine model of traumatic brain injury. Nrf2 anti-oxidant response-element pathway stimulation with consequent negative modulation of NF-κB and NLRP3 inflammasome effects are believed to be the underlying mechanism involved [212]. Multiple structural analogs of sinomenine incorporating A ring alterations have been synthesized and tested. They have been demonstrated to be high efficacy inhibitors of cellular IL-1β release at 10 μg/mL concentration [213].

The actions of colchicine (also an alkaloid) in blunting NLRP3 inflammasome activation have already been described earlier in this article in Section 3.1.9.

#### 3.3.10. Diterpenoid (Oridonin)

Oridonin is the principal biologically effective component of *Rabdosia rubescens*, a commonly used over-the-counter (OTC) herbal root (traditional Chinese Medicine) for the therapy of a multitude of inflammatory conditions. Oridonin has been touted to possess antitumor, anti-inflammatory, as well as pro-apoptotic actions [214,215,216]. Oridonin was originally tested in neoplastic models as it suppresses MAPK or NF-κB stimulation and represses inflammasome-unrelated release of proinflammatory mediators like TNF-α and IL-6 [217,218,219]. Furthermore, oridonin ameliorated sepsis, colitis, as well as neuroinflammation in relevant animal models [220,221,222]. He et al., 2018 [35] unraveled the fundamental mechanisms of anti-inflammatory activity displayed by oridonin. Oridonin was found to selectively repress NLRP3 inflammasome stimulation with no actions on absent in melanoma 2(AIM2) or NLR family CARD domain-containing protein 4(NLRC4) inflammasome activation, LPS-evoked NLRP3, pro-IL-1β expression, and TNF-α generation [35]. Oridonin precisely interacts with the NACHT(NTPases (NAIP, CIIA, HET-E, and TP1)) moiety of NLRP3, and the cysteine 279 on NACHT is a covalent interaction location of oridonin [35]. This covalent binding averts cooperation with NEK7–NLRP3 as well as the consequent NLRP3 inflammasome stimulation. In a murine model, circulating IL-1β concentrations and neutrophilic infiltration were attenuated by oridonin administration (20 mg/kg) following intraperitoneal injection of MSU crystals. Intraperitoneal injection of oridonin (3 mg/kg) every day for 6 weeks in mice given a high-fat diet for 12 weeks decreased fasting and basal glucose concentrations and insulin sensitivity when correlated with control animals. Hence, oridonin produces therapeutic actions on peritonitis, gouty arthritis, and type 2 diabetes in an NLRP3 inflammasome-dependent way [35].

#### 3.3.11. Limonoid Substrate (Fraxinellone)

Fraxinellone, a natural occurring lactone, is isolated from *Dictamnus dasycarpus*, traditional herbal medicine that attenuates inflammatory conditions. Contemporary studies have reported that fraxinellone has a salutary therapeutic action in animal models with inflammatory afflictions. Fraxinellone has proven immunosuppressive activity. Sun et al., 2009 [223] established that fraxinellone mitigated immunologic liver injury with great efficiency and reduced toxicity by specifically engendering the apoptosis of concanavalin A (a lectin)-stimulated T cells, both in vitro as well as in vivo. Fraxinellone also down-regulates inducible nitric oxide synthase (iNOS) and cyclooxygenase-2 (COX2) via the suppression of NF-κB in the RAW 264.7 macrophage cell line resulting in anti-inflammatory effects [224]. A plethora of reports have documented the non-immunosuppressive effects of fraxinellone, which are its insecticidal, antinociceptive, and vasorelaxing properties [225,226,227].

In an acute pancreatitis animal model, fraxinellone inhibited NF-κB and attenuated inflammatory effects in macrophages, inhibited NLRP3 inflammasome stimulation, and IL-1β as well as IL-18 expression. Earlier studies have documented that intervention with fraxinellone ameliorated colitis severity in murine models via suppression of NLRP3 inflammasome activation [228,229,230].

### 3.4. Miscellaneous Agents

#### 3.4.1. Inhibitors of Kinase Having Indirect NLRP3 Inhibitory Effects

It has been established that various kinases modulate NLRP3 inflammasome constituents, and therefore, NLRP3 complex generation [231]. Disentangling and comprehending this complete set of inflammasome protein kinases encoded in its genome (kinome) network is at inception. There have been intense drug discovery endeavors in cancer chemotherapy employing kinase inhibitors; hence, there is a preexisting propitious infrastructure for identifying cutting-edge leads for modulating inflammasomes.

In 2013, Hara et al., 2013 [232] established that Syk and Jnk kinases were imperative for activation of the NLRP3 inflammasome. Syk kinase is believed to phosphorylate apoptosis-associated speck-like protein (ASC), a step that is vital for oligomerization [233]. Employing a chemical biology-driven approach, Hara et al., 2013 [232] put to the test a number of kinase inhibitors to determine which ones effectively prevented IL-1β release. In this study, R406 (the biotransformed effectual form of prodrug fostamatinib) and BAY61-3606 were demonstrated as Syk kinase suppressors [232]. Incidentally, fostamatinib had already made an entry into clinical development as a therapeutic intervention for countering rheumatoid arthritis. However, post-phase IIb clinical trials were abandoned because primary and secondary trial endpoints were not accomplished [234]. Notably, the covalent alterer of NLRP3 inflammasome constituents, 3,4-methylenedioxy-β-nitrostyrene (MNS), is also an established Syk kinase inhibitor and inhibits NLRP3 inflammasome [24]. Hara et al., 2013 [232] also ascertained that AP600125 and peptidic TAT-JI_TIP Jnk inhibitors were potent inflammasome inhibitors [232]. There exist many other documented and commercially available Syk and Jnk kinase inhibitors (nilvadipine [235], TAK659 [236], bentamapimod [237], tanzisertib [237]), which may now be tested for a probable inhibitory activity for combating inflammasome activation.

A multitude of various kinases has been documented to control NLRP3 inflammasome build-up in the literature. They are protein kinase R(double-stranded RNA–dependent protein kinase (PKR)) [238,239], Lyn (which is a representative of the Src lineage of intracellular membrane-associated tyrosine kinases (SFK) (Lyn)) [240], Bruton’s tyrosine kinase (BTK) (which plays a crucial role in B-cell development) [241], TAK1-JNK(Mitogen-activated protein kinase kinase kinase 7 (MAP3K7), also christened as TAK1, is an enzyme that is encoded in humans by the *MAP3K7* gene; c-Jun N-terminal kinases (JNKs) also exist in the mitogen-activated protein kinase group, and are receptive to stressors, such as cytokines, ultraviolet irradiation, heat shock, and osmotic shock, while also contributing to T cell differentiation as well as the cellular apoptosis cascade) [242], phosphatidylinositol-3-kinase (PI(3)K) [243], death-associated protein kinase (DAPK) [244], IL-1 receptor-associated kinase (IRAK1/4) [245,246], proline-rich tyrosine kinase 2 (Pyk2) (non-receptor protein tyrosine kinase) [247], and never in mitosis A(NIMA)-related kinase 7(NEK7) (a Ser/Thr mitotic kinase) [248].

Bruton’s tyrosine kinase (BTK) is an indispensable constituent of the NLRP3 inflammasome, in which BTK tangibly associates with ASC and NLRP3. Pharmacological or genetic methods leading to BTK suppression critically blunts NLRP3 inflammasome activation. The FDA-approved BTK inhibitor ibrutinib (PCI-32765) has been documented to limit infarct volume progression and neurological injury in a murine brain ischemia/reperfusion model. Ibrutinib suppresses IL-1β maturation by blunting caspase-1 stimulation in macrophages and neutrophils, which infiltrate in the ischemic brain infarct. There is a strong indication that BTK is mandatory for NLRP3 inflammasome stimulation, and therefore is anticipated to be a potential therapeutic target in cases of ischemic stroke [241].

TAK1 has been demonstrated to modulate downstream cytokine expression like TNF. Due to this regulatory effect on TNF, TAK1 has assumed importance as an interesting moiety for therapeutic intervention not only in TNF-associated maladies like autoimmune conditions (of the likes of rheumatoid arthritis, lupus, and IBD) but also other cytokine-related dysregulations like chronic pain and cancer [249]. With the availability of novel selective TAK1 inhibitors, scientific research has been undertaken to probe the therapeutic effectiveness of TAK1 targeted therapeutics. The specific TAK1 inhibitor takinib tested at Duke University ameliorated rheumatoid arthritis-like disorder in CIA murine animal model of human joint inflammatory conditions [250]. Moreover, pharmacological suppression of TAK1 has been demonstrated to markedly attenuate inflammatory cytokine levels, most notably TNF levels [251].

Since wortmannin and LY294002 are nonspecific PI3K inhibitors and also inhibit a multitude of unrelated proteins at higher concentrations, they are unlikely to be safe for therapeutic use. The current focus of pharmaceutical firms is to develop PI3K isoform-specific inhibitor agents. As of January 2019, FDA approval has come for three PI3K inhibitors for regular human application in clinical settings, viz., the PIK3CD inhibitor idelalisib (July 2014, NDA 206545), the dual PIK3CA and PIK3CD inhibitor copanlisib (September 2017, NDA 209936), and the dual PIK3CD and PIK3CG inhibitor duvelisib (September 2018, NDA 211155). Concurrent abrogation of multiple signaling pathways such as PI3K, MAPK or PIM has been recommended as an encouraging pharmacotherapeutic strategy to combat cancer. This strategy may prove to be of greater interest over the monotherapeutic strategy by thwarting compensatory signaling, decelerating the buildup of resistance, and potentially permitting decrement in drug dosing [252,253,254,255,256,257].

Of late, NEK7 has been documented to be associated with NLRP3 inflammasome activation via several signaling pathways, like ROS signaling, potassium efflux, lysosomal destabilization, as well as NF-κB signaling [258]. Shi et al., 2016 [259] dwelled upon the NLRP3-NEK7 axis in great depth, demonstrating that NEK7 happens to be a selective upstream modulator of NLRP3 inflammasome stimulation. NEK7 has been shown to interact directly with the NLRP3 leucine-rich repeat segment in a kinase-unrelated fashion, and this interaction is mandatory for the aggregation of inflammasome components. NEK7 is quite selective since it interacts with neither NLRC4 (another LRR segment harboring inflammasome) and AIM2 (lacking an LRR segment) stimulation nor toll-like receptor (TLR) effects. Nevertheless, the modulatory effects of NEK7 as mitotic kinase and NLRP3 modulator are mutually exclusive, i.e., mitosis and NLRP3 inflammasome stimulation cannot take place concurrently. Comprehending and disturbing the NEK7-NLRP3 cooperation could present a unique strategy to target NLRP3 activity. Researchers have come to ground-breaking conclusions on the modulation of gene transcription or protein expression effects of NLRP3 inflammasome signaling cascade operating through NEK7 effects in NLRP3-associated maladies, like tumors, inflammatory diseases, and autoimmune diseases, such as squamous cell carcinoma of head and neck, hepatocellular carcinoma, diabetic retinopathy, systemic lupus erythematosus, gout, atherosclerosis, type 2 diabetes, metabolic syndrome, age-related macular degeneration, Alzheimer’s disease, multiple sclerosis, and inflammatory bowel disease [260,261].

Scant information is available regarding the interaction of kinases with NLRP3; some other inflammasomes have been even less researched. Only if we can comprehend and dissect the kinase signaling networks encompassing these processes, their entire promise can be utilized, and pertinent kinase inhibitors may be ushered or repurposed into therapeutics as anti-inflammatory agents.

#### 3.4.2. Caspase Inhibitors (Pralnacasan (VX-740), Emricasan, VX-765, Thalidomide)

The terminal aim of the inflammasome is stimulation of caspase-1 in a way that leads to cytokine generation. A feasible therapeutic modality would be to suppress caspase-1 straight; there are a plethora of caspase-1-targeted molecules in the scientific literature [36,37]. Pralnacasan (VX740), emricasan, and VX765 are such molecules which could inhibit caspases. Human caspases 4 and 5 (murine ortholog is caspase-11) are also believed to be crucial drug targets. Caspases collaborate with LPS by their CARD domain to evoke protease function activation [38]; this has been christened as the non-canonical inflammasome signaling pathway. In spite of significant research, caspase inhibitors are yet to make an impact in the clinic scenario. There are vital issues apart from specificity challenges; unacceptable toxicity and unfavorable pharmacokinetics have been the bane of available caspase inhibitors. However, an avid interest persists in developing these molecules further, and it is fervently hoped that medication will emanate out of this sustained endeavor, probably coupled with unique drug delivery systems [37].

#### 3.4.3. Ion Channels, Reactive Oxygen Species and Lysosomal Destabilization

There is substantial documentation in the scientific literature suggesting the role of Ca^2+^ in inflammasome provoking. An increment in cellular Ca^2+^ can evoke NLRP3 activation by processes that are still nebulous; on the other hand, calcium channel inhibition precludes NLRP3 stimulation [262,263]. Nilvadipine, which is an inhibitor of calcium channels in addition to being a documented Spleen tyrosine kinase (Syk) inhibitor, may be an alluring molecule to inhibit NLRP3 inflammasome stimulation. Treatment with Nilvadipine ameliorated the TBI-induced inflammatory response in aged mild traumatic brain injury (r-mTBI) animals and notably augmented spatial memory. This was a preclinical study concentrating on the intervention of repetitive r-mTBI in the elderly, and the outcomes favor a therapeutic promise of nilvadipine for sequelae of mTBI [264]. Nilvadipine has the capacity to be of benefit during multimodal Alzheimer’s Disease (AD) treatment by its effects mediated by the unique target of Syk; and a Phase III clinical trial of Nilvadipine in AD has newly been fruitfully wrapped up in Europe [265] (ClinicalTrials.gov: NCT02017340).

Zhou et al., 2010 [266] established that the production of mitochondrial reactive oxygen species (ROS) could evoke NLRP3 inflammasome stimulation, and exposure of macrophages to NLRP3 stimulators led to NLRP3 engagement with mitochondria-associated ER membranes (MAMs) and consequently ASC adaptor recruitment which is crucial for inflammasome generation. ROS generation may follow lysosomal rupture (which can lead to NLRP3 stimulation), leading to activation of calcium-reliant ion channels. Calcium-reliant protein kinases (CaMkII) are also evoked upstream to the TAK1-JNK signaling cascade, culminating in NLRP3 stimulation [252]. Anti-oxidants and radical scavenging agents can impede the action of ROS in evoking the inflammasome. There are many anti-oxidants present in dietary natural products. Protease cathepsin B (which is released upon lysosomal injury) is known to stimulate NLRP3 inflammasome activation. Cathepsin B inhibitors like E-64 [267] or Ca-074 [267] could act as potential NLRP3 inflammasome inhibitor agents.

#### 3.4.4. Type 1 Interferon (IFN) and IFN-Beta

Type I interferons (IFNs), like IFN-α and IFN-β, have been employed to suppress the NLRP3 and other inflammasomes for many auto-immune and auto-inflammatory afflictions. These maladies include multiple sclerosis, systemic-onset juvenile idiopathic arthritis arising out of gain-of-function NLRP3 mutations, rheumatic maladies, and familial-type Mediterranean fever [268,269,270,271,272]. Type I IFNs are generated by macrophages and dendritic cells (DCs) due to extracellular microbial (bacterial and viral) and irritant environmental stimuli [273]. The type I IFN receptor (IFNAR) is a constituent of the TLR lineage, being made up of two subunits IFNAR1 and IFNAR2. IFNAR stimulation concerns a multitude of proteins, comprising Janus kinases, tyrosine kinase 2, and various types of signal transducers and activators of transcriptions (STATs). However, the mechanisms by which type I IFNs interact with the NLRP3 inflammasome culminating in the release of IL-1β and IL-18 remain fuzzy [268]. Progress in this area has been on IFN treatment against multiple sclerosis in patients and EAE in mice, since type I IFN intervention has been employed as the preferred or conventional intervention for multiple sclerosis for the last 15 years [269].

Malhotra et al., 2015 [271] stratified 97 multiple sclerosis patients treated with IFN-β therapy into responders and non-responders on the basis of clinico-radiological features after 12 and 24 months of therapy. Levels of NLRP3 protein and IL-1β levels were notably attenuated among responders suffering from relapsing-remitting multiple sclerosis rather than non-responders. Guarda et al., 2011 [268] demonstrated that IL-1β generation by primary monocytes was less in multiple sclerosis patients administered IFN-β in comparison to healthy individuals, bolstering the worth of IFN-β intervention. Research on murine bone marrow-derived macrophages by Guarda et al., 2011 [268] demonstrated that IFN-β suppressed IL-1β generation via primarily two processes. The first mechanism is that STAT1 transcription factor phosphorylation results in inhibition of NLRP1 and NLRP3 inflammasomes, with consequent repression of caspase-1-reliant IL-1β maturation. In the other pathway, type I IFNs evoke IL-10 secretion through a STAT-reliant process, and the IL-10 engages itself as an autocrine molecule to attenuate levels of pro-IL-1α and pro-IL-1β through a process based upon STAT3 signaling.

Type I IFN therapy is ineffective for all varieties of multiple sclerosis, and the NLRP3 inflammasome seems to be the primary determining factor. Inoue et al., 2012 [270] carried out experiments on murine primary macrophage cultures as well as EAE mice and concluded that IFN-β therapy works only in those cases where the NLRP3 inflammasome is involved directly in the disease process. Moreover, their studies elaborated that repression of IFNAR stimulation could be achieved by engaging the suppressor of cytokine signal 1 (SOCS1), which in turn suppressed Rac1 stimulation and ROS production, with consequent NLRP3 inflammasome inhibition and less intense EAE.

These experiments bring out the effectiveness of type I IFN intervention and the necessity for prospective research for unravelling the processes of NLRP3 inflammasome repression. This work has the potential to enhance clinical methodologies to manage multiple sclerosis as well as various autoimmune and auto-inflammatory afflictions.

#### 3.4.5. Autophagy Inducers (Resveratrol, CB2R Agonist)

A plethora of ways for inhibiting the NLRP3 inflammasome have come into vogue lately. Autophagy, a self-defensive lysosome-associated catabolic process, is now known to repress the NLRP3 inflammasome, leading researchers to probe the benefit of autophagy-inducing interventions [274]. Chang et al., 2015 [209] determined that the phytopolyphenolic agent resveratrol, documented as an autophagy inducer, represses mitochondrial injury in macrophages with consequent NLRP3 inflammasome inhibition and NLRP3 inflammasome-related IL-1β secretion and pyroptosis. Abderrazak et al., 2015 [155] demonstrated that arglabin blunts the generation and liberation of IL-1β and IL-18 by the NLRP3 inflammasome in a concentration-reliant fashion in ApoE−/− mice fed a high-fat diet. The attenuated IL generation implies less intense atherosclerosis. Arglabin produces its actions in macrophages by evoking autophagy and by attenuating inflammation and cholesterol levels.

Cannabinoid receptor 2 (CB2R) has been documented as a therapeutic target in inflammation-associated afflictions [275]. Shao et al., 2014 [274] has explained the reason for stimulation of the anti-inflammatory CB2R leading to repression of NLRP3 inflammasome kindling and stimulation (in a manner analogous to autophagy induction) in murine BV2 microglia treated with LPS and ATP as well as in a murine model of EAE. CB2R stimulation attenuates the intensity of murine EAE. Hence, CB2R agonists like HU-308 may be promising effective agents for intervening in NLRP3 inflammasome-related diseases via autophagy induction.

#### 3.4.6. MicroRNAs (223, 155, 377, 133a-1, etc.)

MicroRNAs may hand out one more opportunity for suppressing inflammasomes. These endogenous non-coding RNAs are 20–23 nt long and associate with the 3 untranslated parts (3 UTR) of protein-coding mRNAs to control their translation [276,277]. MicroRNA-223 attaches to a conserved location in the 3 UTR of the NLRP3 transcript, inhibiting protein synthesis and consequently blocking NLRP3 inflammasome activation and IL-1β generation [277,278,279]. A lack of microRNA-223 is associated with neutrophilia, spontaneous lung inflammation, and heightened proclivity to an endotoxin challenge in mice [280,281]. A few other microRNAs have been documented to be players in the stimulation of the NLRP3 inflammasome, such as microRNA-155, microRNA-377, and microRNA-133a1. Decreasing the concentrations of such agents may prove beneficial in the intervention of inflammasome-associated disorders [282,283,284].

#### 3.4.7. Hydrogen Sulphide

Hydrogen sulfide (H_2_S), as the third gasotransmitter, has been demonstrated to be efficacious in the preventive intervention of inflammation. Also, the NLRP3 inflammasome is a crucial component in the pathogenesis of dextran sulfate sodium (DSS)-induced colitis. Hence, research has been conducted in order to assess if H_2_S produces an anti-inflammatory activity in DSS-induced colitis by acting on the NLRP3 inflammasome. Data showed that DSS-induced colitis is attenuated by H_2_S, ameliorating colon length shortening as well as colonic pathological injuries. The cytokine levels of TNF-α, IL-1β, and IL-6 in colon samples were also markedly decreased by H_2_S. Additionally, H_2_S notably inhibited the expression of NLRP3 and cleaved caspase-1 (p20) in colons from DSS-induced colitis mice. Furthermore, CSE−/− mice had a proclivity towards DSS-induced colitis when correlated with wild-type (WT) mice. Results also indicated that H_2_S dose-dependently represses the stimulation of NLRP3 inflammasome in bone marrow-derived macrophages (BMDMs) by decrementing the cleavage of caspase-1 and IL-1β secretion. Moreover, the suppressive action of H_2_S arises out of a reduction in reactive oxygen species (ROS) formation as well as by disturbing nuclear erythroid 2-related factor-2 (Nrf2) stimulation. In summary, there is confirmation that H_2_S produces its salutary actions in DSS-induced mouse colitis, at least in part by suppressing the activation of the NLRP3 inflammasome signaling pathway [285].

## 4. Conclusions

The intensive role of the inflammasome in masterminding innate immune responses (arising out of microbial infections and non-infectious diseases) has been proven beyond doubt by the occurrence of several heritable and acquired maladies which stem out of dysregulated NLRP3 inflammasome activation and the effectiveness of antagonists of IL-1β or its receptor for intervention in many of these disease conditions. NLRP3-induced pyroptosis and IL-1β/18 secretion are associated with numerous maladies. The degree to which NLRP3 inflammasome stimulation confers towards pyroptosis is still unclear, but NLRP3 activation does lead to pyroptosis, which consequently can inflict severe damage to crucial body organs [286]. Currently, NLRP3-linked afflictions may be ameliorated by agents which could abrogate IL-1β, such as counteracting IL-1β antibody canakinumab, recombinant IL-1 receptor antagonist anakinra, and the soluble camouflage IL-1 receptor, rilonacept. These biological molecules have been employed to manage cryopyrin-associated periodic syndromes (CAPS) as well as various maladies related to IL-1β [287]. Apart from NLRP-3 generated IL-1β, other cytokines like IL-18 may also assist with the development of the NLRP3-related afflictions [288,289]. Other inflammasomes or inflammasome-independent pathways can also lead to IL-1β production; hence, IL-1β inhibitors can only lead to non-intentional immunosuppressive actions. Thus, pharmacological inhibitors focusing on NLRP3 inflammasome inhibition would be a preferred alternative for combating NLRP3-mediated maladies. NLRP3-mediated pyroptosis has been documented by a plethora of contemporary studies as a crucial system adding to the NLRP3 inflammasome linked disease processes [290,291]. Documentation surfacing has noted gasdermin D (GSDMD) as a controlling protein culpable for pyroptosis [292,293], making it an enticing therapeutic target for ameliorating NLRP3-evoked pyroptosis associated disorders. Since knowledge of the structure and components of the NLRP3 inflammasome has become available, forthcoming studies should harness this lead and effect progress of development of direct NLRP3 inflammasome inhibitors endowed with increased exactitude and potency. Moreover, nanobodies (Nbs) are lately being scrutinized comprehensively as therapeutics owing to their high precision, stability, and low propensity to induce side effects [294,295]. It can be foreseen that Nbs may also be assessed for inhibiting NLRP3 inflammasome activation. Significant strides have been undertaken to unravel the NLRP3 inflammasome structure, the processes leading to its activation, and its role in the initiation and evolution of various disorders. Moreover, a multitude of small molecules as NLRP3 inflammasome inhibitors have been documented in the research literature, and a few of these leads have highlighted admirable therapeutic prowess. However, none of them has been approved by Food and Drug Administration (FDA) or other drug regulatory agencies. Contemporary research must continuously concentrate on the development of specific, small-molecule NLRP3 inflammasome activation inhibitors with enhanced pharmacokinetic characteristics, the ability to permeate efficiently across the blood–brain barrier and cell membranes, and it must be affordable too. Undoubtedly, the ongoing contemporary characterization of clinical inflammasome activators and blockers will spur interest in acquiring deeper insights into inflammasome-driven autoinflammatory mechanisms from a futuristic point of view. This is bound to widen therapeutic modalities for patients suffering from NLRP3-mediated metabolic and neurodegenerative diseases as well as certain cancers, where there is a tremendous unmet therapeutic need [296]. Finally, an appreciation of the molecular biology in connection with inflammasome priming and activation facilitates the prediction that an array of nutraceuticals could possibly confer salutary clinical promise for impeding inflammasome activity—antioxidants such as phycocyanobilin, phase 2 inducers, melatonin, and N-acetylcysteine, the AMPK activator berberine, glucosamine, zinc, and various nutraceuticals which ramp up production of hydrogen sulfide. Complex nutraceuticals or functional foods consisting of several of such compounds may find value in the obviation and modulation of a great variety of medical disorders [297].

## Figures and Tables

**Table 1:** Some inflammatory diseases with their targets (involving inflammasome signaling components) for possible therapeutic intervention.

Disease	Targets in Inflammasome Signaling Cascade	Therapeutic Molecule
Acute Myocardial Infarction	NLRP3	Colchicine
Type 2 Diabetes Mellitus	NLRP3(indirect action)	Metformin, Glyburide
	IL-1β	Rilonacept
Rheumatoid Arthritis	IL-1 Receptor	Anakinra
	Caspase-1	Pralnacasan(VX-740)
	P2X7	AZD9056, CE-224535, GSK 1482169
Muckle–Wells Syndrome	Caspase-1	Emricasan(VX-765)
	IL-1β	Canakinumab
Gout	IL-1β	Rilonacept
	Xanthine Oxidase(XOD)	Allopurinol
Systemic Lupus Erythematosus	NFκB (IKKβ kinase activity)/NLRP3 ATPase	Bay 11-7082
Cryopyrin-Associated Periodic Syndromes(CAPS)	IL-1β	Rilonacept
Inflammatory Bowel Disease(IBD)	IL-18	GSK1070806
Familial Cold Autoinflammatory Syndrome(FCAS)	IL-1β	Canakinumab
Cancer	Caspase-1/NF-κB (IKKβ kinase activity)/NLRP3 ATPase	Parthenolide
B-cell Non-Hodgkin’s Lymphoma	IL-18	GSK1070806

**Table 2:** Targets of some known NLRP3 Inhibitors.

Inhibitor	Target(s)	Documented Mechanism(s)	References
Sulfonylureas			
Glyburide	NLRP3(indirect action)	Abrogation of ASC agglomeration acting downstream of P2X7; Suppression of K_ATP_ channels	[1]
MCC950	NLRP3	NLRP3 inflammasome activation involves a role of its ATPase domain. MCC950 is known to directly target and restrain this ATP-hydrolysis motif in both canonical as well as non-canonical NLRP3 inflammasomes	[2,3]
Glitazones			
CY-09	NLRP3	Effective and direct suppressor of NLRP3 inflammasome with remarkable capability to impede NLRP3 inflammasome activation in vivo in murine models and ex vivo in human cells; blocks NLRP3 ATPase actions	[4]
Substituted 2-pyrazolin-5-ones			
Edaravone	NLRP3	Scavenge reactive oxygen species(ROS) thereby impeding NLRP3-evoked IL-1β processing and release; also known to suppress IL-1β, caspase 1 and NF-kB-reliant NLRP3 inflammation signaling	[5,6]
Arsenic compounds			
Arsenic trioxide(As_2_O_3_)	NLRP3	As_2_O_3_ suppresses NLRP3 inflammasome stimulation and consequent IL-1β and IL-18 release	[7,8]
Alkaloid			
Colchicine	NLRP3	Efficaciously attenuates the expression levels of IL-1β, IL-6 and IL-18 by abrogating NLRP3 inflammasome activation cascade	[9,10]
Biguanide			
Metformin	NLRP3	Adenosine monophosphate-activated protein kinase(AMPK) is known to modulate NLRP3 inflammasome stimulation; decreases the expression of NLRP3 as well as kindling of the NLRP3 inflammasome signaling pathway	[11,12]
GLP-1 analogs			
Liraglutide	NLRP3(hepatic)	Repression of the hepatic NLRP3 inflammasome	[13]
Statins			
Atorvastatin	NLRP3	Conspicuously decrements levels of NLRP3, caspase-1, and IL-1β; also, the NF-κB suppressor attenuate levels of inflammatory cytokines in inflammatory cells. The stimulation of the NF-κB signaling cascade is engaged in NLRP3 inflammasome activity modulation	[14,15]
SGLT-2 Inhibitors(Dapagliflozin, Empagliflozin)-P2Y12 Antagonist(Ticagrelor)			
Dapagliflozin	NLRP3	Extenuates inflammation-evoked renal damage and glomerulosclerosis in diabetic kidneys by ameliorating NLRP3 inflammasome stimulation; AMPK activation	[16]
Empagliflozin	NLRP3	Impedes kindling of NLRP3 inflammasome and decrements downstream inflammatory signaling in the diabetic kidneys	[17]
Ticagrelor	NLRP3	Repress NLRP3 inflammasome stimulation; AMPK activation	[18]
Xanthine oxidase(XOD) enzyme inhibitor			
Allopurinol	NLRP3, XOD	Represses xanthine oxidase(XOD) action and subsequently attenuates generation of uric acid (UA) and reactive oxygen species (ROS), which are known to kindle the NLRP3 pathway	[19,20]
Vinylsulfones			
BAY11-7082	NLRP3, IKK, E2/3 enzymes, PTPs	Leads to cysteine alkylation of NLRP3 inflammasome ATPase domains; represses NLRP3 ATPase actions	[4,21]
Beta-Nitrostyrenes			
MNS	NLRP3	Leads to cysteine alteration of NLRP3 inflammasome ATPase domains; represses NLRP3 inflammasome actions	[22]
Acrylate Derivatives			
INF39	NLRP3	Abrogates NLRP3 inflammasome ATPase actions; represses priming	[23]
Acylhydrazone			
EMD638683	NLRP3	Suppression of NLRP3 and IL-1β expression	[24]
Benzimidazoles			
FC11A-2	NLRP3(indirect effect)	Hampers pro-caspase-1 autocleavage; impedes IL-1beta/18 secretion	[25,26]
Sulfonylnitriles			
Dapansutrile(OLT1177)	NLRP3	Abrogates NLRP3 inflammasome ATPase actions; suppresses NLRP3 inflammasome stimulation	[27,28]
Benzoxathiole Derivatives			
BOT-4-one	NLRP3	Akin to various covalent modulators that repress NLRP3, this agent blunts its ATPase activity; inhibits priming	[29,30,31]
Tryptophan Derivative			
Tranilast	NLRP3	Interacts with NACHT segment of NLRP3 to abrogate NLRP3-NLRP3 and NLRP3-ASC association	[4,32]
Natural Products			
BHB	NLRP3(indirectly)	Abrogation of outward movement of K+ with consequent decrement in ASC agglomeration and IL-1beta/18 release	[33]
Parthenolide	NLRP1 & 3, Caspase- 1, NF-kB, IKKB kinase activity	Alkyl modification of cysteine moieties present in ATPase segments of NLRP3 and caspase-1; abrogates NLRP3 ATPase actions	[34]
Oridonin	NLRP3	Selectively represses NLRP3 inflammasome stimulation; associates with cysteine 279 residue of NLRP3 and abrogates NLRP3-NEK7 association	[35]
Caspase Inhibitors			
Pralnacasan(VX-740)	Caspase-1	Covalent alteration of catalytic cysteine moiety in caspase-1 active site with consequent abrogation of caspase-1 effects and splitting of pro-IL-1Beta/18	[36,37,38]
Emricasan(VX-765)	Caspase-1	Covalent alteration of catalytic cysteine moiety in caspase-1 active site with consequent abrogation of caspase-1 effects and splitting of pro-IL-1Beta/18	[36,37,38]

## Data Availability

Not applicable.
